# Grapevine cell response to carbon deficiency requires transcriptome and methylome reprogramming

**DOI:** 10.1093/hr/uhae277

**Published:** 2024-09-28

**Authors:** Margot M J Berger, Virginie Garcia, Nathalie Lacrampe, Bernadette Rubio, Guillaume Decros, Pierre Pétriacq, Amélie Flandin, Cédric Cassan, Ghislaine Hilbert-Masson, Sophie Colombié, Rossitza Atanassova, Philippe Gallusci

**Affiliations:** Ecophysiologie et Génomique Fonctionnelle de la Vigne (EGFV), University of Bordeaux, Bordeaux Sciences Agro, INRAE, ISVV, Villenave d’Ornon, France; Ecophysiologie et Génomique Fonctionnelle de la Vigne (EGFV), University of Bordeaux, Bordeaux Sciences Agro, INRAE, ISVV, Villenave d’Ornon, France; Plantes et Système de culture horticoles (PSH), INRAE, UMR Qualisud, Université Avignon, Avignon, France; Ecophysiologie et Génomique Fonctionnelle de la Vigne (EGFV), University of Bordeaux, Bordeaux Sciences Agro, INRAE, ISVV, Villenave d’Ornon, France; Univ. Bordeaux, INRAE, UMR1332 BFP, 33882 Villenave d’Ornon, France; Univ. Bordeaux, INRAE, UMR1332 BFP, 33882 Villenave d’Ornon, France; Bordeaux Metabolome, MetaboHUB, PHENOME-EMPHASIS, 33140 Villenave d’Ornon, France; Univ. Bordeaux, INRAE, UMR1332 BFP, 33882 Villenave d’Ornon, France; Bordeaux Metabolome, MetaboHUB, PHENOME-EMPHASIS, 33140 Villenave d’Ornon, France; Univ. Bordeaux, INRAE, UMR1332 BFP, 33882 Villenave d’Ornon, France; Ecophysiologie et Génomique Fonctionnelle de la Vigne (EGFV), University of Bordeaux, Bordeaux Sciences Agro, INRAE, ISVV, Villenave d’Ornon, France; Univ. Bordeaux, INRAE, UMR1332 BFP, 33882 Villenave d’Ornon, France; Bordeaux Metabolome, MetaboHUB, PHENOME-EMPHASIS, 33140 Villenave d’Ornon, France; UMR CNRS 7267 EBI-Ecologie et Biologie des Interactions (EBI), Equipe Sucres et Echanges Végétaux-Environnement (SEVE), University of Poitiers, Poitiers, France; Ecophysiologie et Génomique Fonctionnelle de la Vigne (EGFV), University of Bordeaux, Bordeaux Sciences Agro, INRAE, ISVV, Villenave d’Ornon, France

## Abstract

Sugar limitation has dramatic consequences on plant cells, which include cell metabolism and transcriptional reprogramming, and the recycling of cellular components to maintain fundamental cell functions. There is however no description of the contribution of epigenetic regulations to the adaptation of plant cells to limited carbon availability. We investigated this question using nonphotosynthetic grapevine cells (*Vitis vinifera*, cv Cabernet Sauvignon) cultured *in vitro* with contrasted glucose concentrations. Sugar depletion in the culture medium led to a rapid cell growth arrest and a major metabolic shift that include the depletion in soluble sugar and total amino acids and modulation of the cell redox status. Consistently, flux modeling showed a dramatic slowdown of many pathways required for biomass accumulation such as cell wall and protein synthesis. Sugar depletion also resulted in a major transcriptional reprogramming, characterized by the induction of genes involved in photosynthesis, and the repression of those related to sucrose mobilization or cell cycle control. Similarly, the epigenetic landscape was deeply modified. Glucose-depleted cells showed a higher global DNA methylation level than those grown with glucose. Changes in DNA methylation mainly occurred at transposable elements, and at genes including some of those differentially expressed, consistent with an important role for methylation to the adaptation of cells to limited sugar availability. In addition, genes encoding histone modifiers were differentially expressed suggesting that additional epigenetic mechanisms may be at work in plant cells under carbon shortage.

## Introduction

Epigenetics is defined as the study of heritable and/or stable changes in gene expression that occur without modifications of the DNA sequence [1]. Epigenetic regulations involve different mechanisms such as post-translational modification of histones, small RNA production and DNA methylation, and play important roles in plant development and adaptation to the environment [2, 3] In plants, methylation of the fifth carbon of cytosine can occur in the symmetrical CG and CHG and nonsymmetrical CHH (H = A, C or T) sequence contexts. Establishment of DNA methylation in all sequence contexts is performed by the RNA directed DNA methylation (RdDM) pathway that requires the domain rearranged methyltransferases 1, 2 (DRM1/2), DRD1 and 24 nt-long small RNAs, and by the chromomethylase 2 (CMT2) associated with decrease in DNA Methylation (DDM1) for CHH methylation in constitutive heterochromatic regions. Methylation at hemi methylated CG sites, generated during DNA replication, relies on the activity of Methyltransferase 1 (MET1) together with variant in methylation (VIM) 1, 2, and 3, and at CHG sites on CMT3. Methylation of the newly synthesized DNA strands at nonmethylated CHH sites is mediated by both the RdDM pathway and CMT2 [4]. DNA methylation can be passively lost during cell divisions, or actively removed by DNA demethylases in a base–excision repair process [5].

Links between epigenetic regulations and metabolism were recently evidenced in animal systems [6]. Epigenetic modifications, both DNA methylation and histone post-translational modifications (HPTMs) are mediated by enzymes, that require metabolic precursors including acetyl-coenzyme A (Acetyl-CoA) or S-adenosyl-methionine (SAM), and cofactors (nicotinamide adenine dinucleotide; NAD+, acetyl-CoA). Epigenetic processes are therefore, intimately connected to cell metabolism [7]. This is further indicated by evidence of an interplay between sugar availability and epigenetic regulations in eukaryotes [8, 9]. Recent works in yeast and yellowfin seabream have shown that starving is associated with metabolic imbalance and transcriptomic reprogramming. Main effects include the reduced expression of genes involved in cell growth while promoting those involved in carbon remobilization (fatty acid oxidation, gluconeogenesis) [10, 11]. In addition, while in yeast there is growing evidence of an interplay between the genome wide distribution of histone acetylation and transcriptomic changes following sugar starvation [10], in yellowfin seabream modification of the global DNA methylation level and distribution is considered as an important consequence of starvation [11]. In plants, carbon depletion is also associated with several cellular responses. On the one hand, starvation promotes protein, amino acids and lipid degradation, cell wall, starch and sucrose degradation, autophagy, photosynthesis, gluconeogenesis, transporters, and trehalose metabolism. On the other hand, carbon deprivation inhibits amino acids and protein synthesis, cell wall biogenesis, nucleotide metabolism, TCA cycle and glycolysis. The change of the cell energy status requires two antagonistically acting metabolic sensors—sucrose-non-fermenting-related kinase 1 (SnRK1) and target of rapamycin (TOR) kinase, which orchestrate the cell energy homeostasis, growth, and survival throughout transcriptional and epigenetic regulations. Carbon starvation triggers the formation of SnRK1-C/S1-bZIP to enhance histone acetylation, or the direct interaction of SnRK1 with JMJ705 to decrease H3K27me3 levels, thus activating the expression of starvation-responsive genes to maintain intracellular energy homeostasis [12, 13]. In addition, recent work in Arabidopsis has shown that rapamicyn, an inhibitor of the TOR kinase, affects DNA methylation at genes involved in carbon and amino acid metabolism, suggesting that the glucose-TOR effect is mediated in part by DNA methylation [14]. Furthermore, mutation or pharmacological approaches impairing the synthesis of metabolic precursors necessary for epigenetic regulations, such as acetyl-CoA [15] or SAM, have important impacts on epigenomes, gene expression and plant phenotypes [16, 17].

In this work, we used carbon depleted heterotrophic grapevine cells as a way to investigate possible links between cell metabolism and DNA methylation control.

The consequences of carbon depletion on cell growth, metabolic profiles, and metabolic fluxes were described. We also analyzed the molecular response of cells by determining changes in gene expression profiles and DNA methylation landscapes. Correlative analyses of these multi-omics data sets allowed demonstrating tight interactions between cell primary metabolism, and gene expression and DNA-methylation reprogramming, thus emphasizing the central role of epigenetic regulations in the response of cells to sugar starvation.

## Results

### Sugar depletion causes a rapid cell growth arrest and metabolic shift

**Figure 1:**
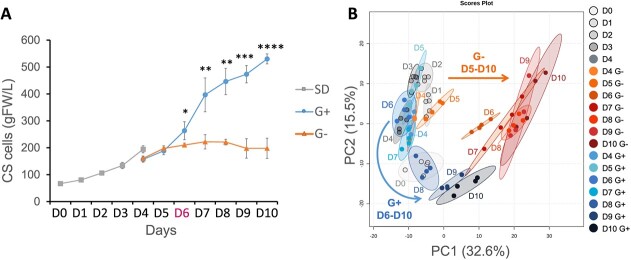
Sugar depletion triggers rapid growth arrest and metabolic drift in grapevine cells. (A) Cabernet Sauvignon cell growth expressed in gram fresh weight (gFW/L) in SD (from day 0 to 4, square) and in glucose rich (G+ circle) or glucose poor (G-, triangle) subculture from D4 to D10 (*n* = 4). Vertical bars are confidence interval (*n* ≥ 4). **(B)** Principal component analysis (PCA) score plots (*n* = 4) of 1719 LCMS-based metabolic signatures under SD, G+ or G− conditions. Maximal variance explained by each PC axis is indicated in brackets. Arrows represent the direction of metabolomic evolution in the PCA. SD (D0–D4): grey shades; G+ (D4–D10): blue shades; G− (D4–D10): orange shades. Vertical bars represent CI (*n* = 8). Stars indicate the level of significance of G+ and G− sample comparison (^*^P ≤ 0.05, ^**^P ≤ 0.01, ^***^P ≤ 0.001, ^****^P ≤ 0.0001).

After a latent phase of 3 days, Cabernet Sauvignon cells (CS) grown in standard conditions (SD, see methods) showed a rapid growth phase from day 3 (D3) to D8 followed by a growth arrest (Fig. S1A). To evaluate the impact of glucose depletion, CS cells were transferred at D4 to either a glucose-rich (G+, 110 mM) or a glucose-poor (G-, 11 mM) medium (Fig. 1A). The G+ cells fresh weight (FW) increased ~3,5-fold from D4 to D10 to reach 530.2 (±18.7) g FW/mL at D10 (Fig. 1A). In contrast, G- cell FW showed a 1.2-fold increase, from 160 (±8.93) g FW/mL at D4 to 197.6 (± 9.61) g FW/mL at D5, with no further significant changes. At D6, G- cells growth was significantly reduced as compared to G+ cells (Fig. 1A), which was associated with a severe reduction of sugar availability in the growing medium (Fig. S2).

The consequences of carbon depletion on grapevine cell metabolism, was investigated using untargeted metabolomics analyses (see methods) each day from D0 to D4, and, after subculturing, from D4 to D10 in G+ and G- conditions (*n* ≥ 4). After filtering of the metabolic signatures obtained from all samples analyzed, 1719 variables were selected as the most reliable (see methods). The PCA of these signatures indicate that samples are grouped at D4 and diverge along the PC1 axis (representing 32.6% of the total variance) at D5 (Fig. 1B). Maximum multivariate separation between samples is evident at D6 and D7, whilst G+ cells converge toward G-cells after D8 and this trend consistent with a restriction in glucose availability under G+ conditions at that time point. The G+ cells separate along the PC2 axis (15.5% of the total variance) as a function of time. Pearson’s correlation clustering (Fig. S3) further confirmed the divergence between samples as dependent to both incubation time and medium composition.

Pairwise comparisons of the metabolic profiles showed that the number of metabolites (*P* < 0.01) differentially accumulated between G+ and G- conditions increased from 1 to 56 at D4 and D5, respectively, then to 285 from D5 to D6, with no further increase at D7 (Table 1). At D8, metabolic differences heightened transiently between conditions as G+ cells began to suffer from carbon depletion as well (Fig. S2), before decreasing again from D8 to D10.

**Table 1:** Carbon depletion triggers a metabolic shift in grapevine cells after 48 h of subculture

**T-test p = 0.01**	**D4 G+ vs G-**	**D5**	**D6**	**D7**	**D8**	**D9**	**D10**
Metabolic features	1	56	285	264	433	213	132

Pairwise statistical analysis of the metabolomic features differing between glucose rich (G+) and glucose poor (G−) conditions estimated from D4 to D10.

Metabolomics profiles of G+ and G− cells at D4, D5, D6, and D7 were further used to extract the most significant 596 signals, in terms of differential accumulation in all samples (P < 0.01) over a total of 1703 detected from D4 to D7 (Fig. S4). Utilizing Pearson’s correlation clustering on the 596 signals resulted in samples classification into four distinct groups that present distinct metabolite accumulation profiles from D5 to D7, demonstrating a progressive separation of G+ and G− samples from D4 to D7 (Fig. S4).

Overall, metabolome analyses reveal that changes in glucose availability generate major metabolic adjustment in heterotrophic cells as early as 24 h after subculture.

### Sugar limitation has a profound impact on the cell primary metabolism and redox state

Quantitative metabolic profiling of soluble sugars, organic acids, proteins, and total amino acids was performed from D0 to D10 in all growing conditions. As observed with untargeted metabolic analysis, most of metabolites measured were differentially accumulated between G+ and G- conditions from D6 (Figs S5 and S6). Main effects of G- condition were a reduction of soluble sugars accumulation in cells, but also changes in the abundance of free amino acids, organic acids, or total proteins. The NADH/NAD balance was also severely reduced in sugar-depleted cells (Fig. S5). These data are consistent with a strong impact of carbon depletion on grapevine cell central metabolism and redox state.

To further determine the consequences of sugar depletion on cell metabolism, experimental data of targeted metabolite analyses and cell components were integrated to constrain a flux model analysis and evaluate the impact of carbon depletion on the cell metabolic fluxes. To perform the flux analysis (Tables S1–S4), the metabolic model was constrained to calculate fluxes as a snapshot each day in all conditions (*i.e.,* in SD, G+ and G− conditions; Fig. S7). Most of the internal fluxes calculated in G− were lower than the ones calculated in G+ at D6 (Fig. S7) including those of glycolysis, oxidative phosphate pathway, and cell wall biosynthesis, and to a lower extend those of respiration, nitrate assimilation, and of the TCA cycle. Interestingly, some fluxes were increased under carbon limitation.

They include fluxes involved in the mobilization of stored compounds, such as cell wall polymers, total protein, as well as the accumulation of lipids and some amino acids (Fig. S7; Table S5).

**Figure 2:**
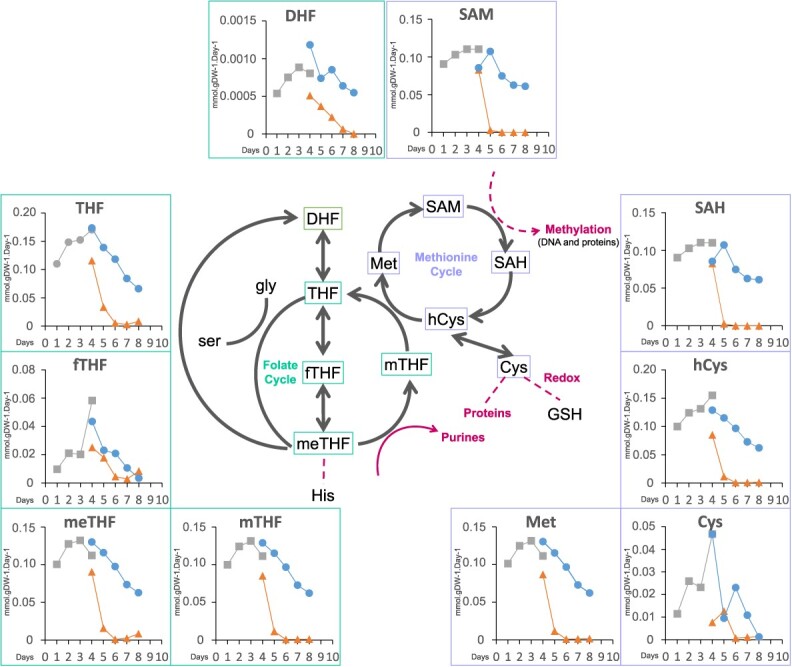
Carbon depletion impacts 1C metabolic fluxes in grapevine cells. Calculated fluxes through metabolites involved in the TetraHydroFolate (THF) and S-Adenosyl-Methionine (SAM) cycle. The x-axis represents time (days of culture), and the y-axis flux values calculated in mmol.gDW^−1^.Day^−1^. From D1 to D4, cells in standard conditions (SD, square); from D4 to D8, cells in glucose-rich culture (G+, circle) and glucose-poor-culture (G-, triangle): SD; blue and orange lines.

We focused on the folate and methionine cycles (the 1C metabolism), as they link primary metabolism to methylation by controlling SAM availability to cells [18]. For the THF and SAM cycles, the calculated fluxes were used to estimate the flux activity for an intermediate metabolite as the sum of fluxes producing (or consuming) this metabolite (Fig. 2; Table S6). Most of the fluxes rapidly dropped down to zero between D4 and D6 under G−, except fluxes through dihydrofolate (DHF), tetrahydrofolate (THF) and 10-formyltetrahydrofolate (fTHF) (Fig. 2, Table S6). By contrast, the SAM fluxes estimated at 0.075 mmol/g DW/day in G+ condition at D6 were reduced to 0.012 × 10^−3^ mmol/g DW/day in G− condition (Fig. 2; Table S6). This suggests that folate synthesis is not completely inhibited under carbon depletion, and that parts of this cycle are prioritized as compared to others. According to the model prediction, folate is *a priori* mainly needed for other DHF-dependent pathways such as nucleotides synthesis (Table S2), rather than for DNA methylation, because SAM cycle fluxes are decreased under carbon depletion. Overall, these results show that sugar depletion impacts 1C metabolic pathways, including those implicated in the synthesis of SAM.

### Genes of several metabolic pathways display differential expression under sugar depletion

RNAseq analyses were performed at D6 in both G+ and G− conditions. After trimming, quality assessment and removal of low-quality reads (see Methods), total cleaned reads, approximately ~24 million reads for each sample, were mapped to the grapevine reference genome (12X.V2; [19], of which on average 90% were uniquely mapped (Table S7). Between 69.7% and 71% of aligned reads overlap known genes. PCA performed using all expressed genes reveals both strong differential behavior between G+ and G− conditions, and low variability between biological replicates (Fig. S8). This observation is also supported by the analysis of the 200 genes showing the highest variation in expression levels between conditions (Fig. S9). Replicates for each condition cluster together in correlative analysis, but are clearly separated between G+ and G− conditions.

A total of 5607 genes were considered differentially expressed (Log2FoldChange >1.0; padj 0.05), among which 2802 were upregulated and 2805 downregulated in G- versus G+ conditions. Gene ontology (GO) overrepresentation analysis showed that upregulated genes are enriched in genes involved in photosynthesis (PS) processes including light harvesting, chlorophyll biosynthesis and electron transport in photosystems, in stress responses (hormones, redox process, defense response), and in metabolite biosynthesis and transport (trehalose, malate, oligopeptide; Fig. S10). By contrast, downregulated genes were enriched in genes involved in translation (ribosome biogenesis, translational elongation), cell division (DNA replication, microtubule-based movements, chromosome segregation), amino acids biosynthesis (serine, threonine, glycine, tyrosine, phenylalanine), and processes related to carbon metabolism (gluconeogenesis, glycolysis, sugars, fatty acids; Fig. S10).

**Figure 3:**
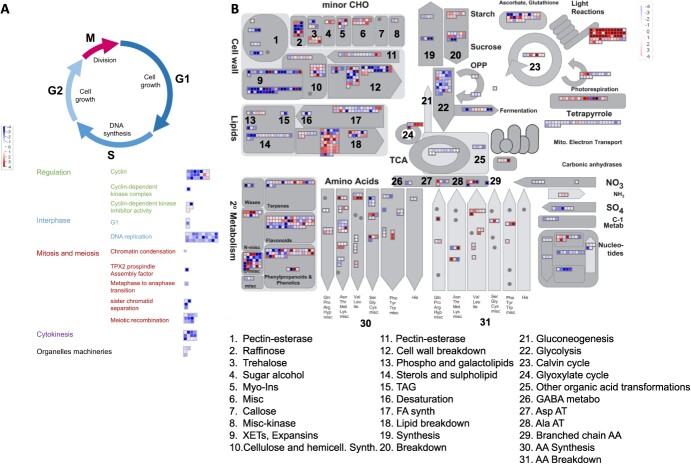
Carbon depletion strongly affects the expression of genes related to cell cycle and metabolic pathways dedicated to carbon mobilization. Mapman visualization of DEGs in grapevine cells in culture in G+ versus G− conditions at D6. **(A)** Cell cycle-related genes. Each square represents a gene. In red: genes more expressed in G− cells; in blue: genes more expressed in G+ condition. **(B)** Mapman representation of metabolic pathways representing DEGs and metabolites in G− versus G+ cells at D6. Each square represents a gene and each circle a metabolite. In red: genes/metabolites more expressed/abundant in G- cells; in blue: genes/metabolites more expressed/abundant in G+ condition.

Using a MapMan representation with both targeted metabolites and RNAseq data (Fig. 3A and B), 89 and 947 out of 5607 DEGs were successfully assigned to cell cycle and various metabolic pathways respectively. Most of DEGs related to cell cycle (82/89) were downregulated at D6 (Fig. 3A), suggesting that the cell growth arrest observed in G- conditions correlates with a stop in cell divisions. When considering DEGs involved in metabolism (Fig. 3B), those associated with cell wall biogenesis (pectin esterases, expansins, and xyloglucane endotransglycosylases (XETs)), amino acid and nucleotide synthesis, glycolysis, TCA cycle, energy metabolism (mitochondrial electron transport), sucrose and starch synthesis, glycolysis and other downstream reactions were downregulated in G− conditions (Fig. 3B, Fig. S11). Inversely, DEGs associated with processes induced by carbon depletion including the mobilization of carbon resources, amino acids, lipids and sucrose, the breakdown of starch and protein together with the glyoxylate cycle, and with the ATG-mediated autophagy pathway [20–22] were up-regulated in G- cells (Figs 3B, S11, Table S8).

Genes involved in 1C metabolism were downregulated at D6 (Fig. 3B), including those related to the synthesis of 5,10-methylenetetrahydrofolate (methylene-THF), a precursor of the tetrahydrofolate cycle (THF cycle) (Fig. S12). Overall, results show that sugar deprivation strongly impacts the expression of genes of the metabolic pathways dedicated to carbon mobilization, nutrient mobilization, and heterotrophic carbon formation and are consistent with previous data obtained on Arabidopsis cells under sugar starvation [23].

### Genes involved in epigenetic processes are differentially regulated under carbon limitation

As a first step to investigate possible effects of sugar depletion on epigenetic regulations, our work focused on the expression of genes involved in epigenetic processes (Table 2). Four DNA-methyltransferases (DNMTs), *VvCMT1*, *VvCMT3*, *VvCMT4*, were down-regulated with a Log2FoldChange (L2FC) ranging between −1.06 and − 1.45 (padj<0.01). Similarly, genes encoding the demethylase (DEMETER Like; DML) *VvDML3* and chromatin remodeler (DECREASE IN DNA METHYLATION 1; DDM1) *VvDDM1* were also down-regulated with a L2FC of −2.60 and − 1.1 (padj<0.01) respectively. Four ARGONAUTE (*VvAGO10a, VvAGO11, VvAGO5, VvAGO2a*), one RNA-dependent-RNA polymerase (*VvRDR1b*), and one DICER-like (*VvDCL2*) genes were also differentially expressed under G− condition. The *VvAGO10a* and *VvAGO11* genes were both down-regulated showing L2FC values of −1.8 and − 1.2 respectively (padj < 0.05), while the four other genes were up-regulated with a L2FC of 1.3 for *VvAGO5* and 1.5 for *VvAGO2a* (padj < 0.05) and a L2FC of 2.3 for both *VvRDR1b* and *VvDCL2* (padj < 0.05; Table 2). We also investigated genes encoding histone modifiers [24] (Table 2), and putative POLYCOMB REPRESSING COMPLEX2 (PRC2; Table 2, Figs S13, S14). Two histone demethylases genes (HDMs, *Vitvi08g00216, Vitvi10g01394*), were downregulated, while three others (*Vitvi10g00053*, *Vitvi15g00950*, *Vitvi16g01410*) were upregulated. All DEGs encoding histone lysine methyltransferases (*HKMTs, VvCLF*/*Vitvi07g01721*, *Vitvi16g00290*, *Vitvi08g01786*, *Vitvi10g00002*, *Vitvi16g02079*) were downregulated in G- conditions with L2FC ranging between −1.08 and − 3.54 (padj < 0.05; Table 2). One histone acetyltransferase gene (HAT, *Vitvi11g00219*), was downregulated in G- condition with a L2FC of −1.31 (padj<0.05). Genes encoding putative POLYCOMB REPRESSING COMPLEX1 (PRC1) (VvDRIP1 *Vitvi07g00031* and VvDRIP3 *Vitvi05g00634*) and PRC2 components, (VvMSI2 *Vitvi03g00147* and VvMSI5 *Vitvi11g00185*) were also differentially expressed under carbon limitation with contrasted behaviors (Table 2).

**Table 2:** Epigenetic modifiers are differentially expressed in grapevine cells under carbon depletion

Name/Function	Gene ID	Diff.Exp^a^	padj
VvDDM1	Vitvi04g01275	−1.1	7.22^E^−22
VvCMT1	Vitvi08g01767	−1.1	3.35^E^−07
VvCMT3	Vitvi06g00102	−1.3	4.58^E^−11
VvCMT4	Vitvi16g00174	−1.5	5.62^E^−38
VvDML3	Vitvi06g01402	−2.6	3.35^E^−27
VvAGO10a	Vitvi05g00574	−1.8	0.04
VvAGO11	Vitvi12g00448	−1.2	3.23^E^−09
VvAGO5	Vitvi06g01378	1.3	9.15^E^−53
VvAGO2a	Vitvi10g01346	1.5	3.78^E^−08
VvRDR1b	Vitvi01g00503	2.3	1.13^E^−59
VvDCL2	Vitvi04g01202	2.3	6.60^E^−118
VvHDM	Vitvi10g00053	1.5	4.68^E^−16
	Vitvi08g00216	−1.1	1.50^E^−10
	Vitvi10g01394	−1.7	1.72^E^−18
	Vitvi15g00950	1.2	1.68^E^−12
	Vitvi16g01410	1.5	3.40^E^−19
VvHKMT	Vitvi07g01721	−1.1	7.41^E^−11
	Vitvi16g00290	−1.7	2.85^E^−05
	Vitvi08g01786	−1.3	0.003
	Vitvi10g00002	−3.3	3.64^E^−25
	Vitvi16g02079	−3.5	2.75^E^−25
VvHAT	Vitvi11g00219	−1.3	8.05^E^−29
VvPRC1	Vitvi07g00031	1.3	3.46^E^−35
	Vitvi05g00634	−2.2	1.01^E^−20
VvPRC2	Vitvi07g01721	−1.1	7.41^E^−11
	Vitvi03g00147	−1.6	3.19^E^−26
	Vitvi11g00185	−1.1	2.45^E^−08
^a^Log2FoldChange			

Grapevine epigenetic modifiers differential expression in G- cells. DDM1: DECREASED DNA METHYLATION 1; CMT: CHROMOMETHYLASE; DML: DEMETER-LIKE; AGO: ARGONAUTE; RDR: RNA-DEPENDENT RNA POLYMERASE; DCL: DICER-LIKE; HDM: Histone Demethylase; HKMT: Histone-Lysine-Methyl-Transferase; HAT: Histone Acetyl-Transferase; PRC: Polycomb Repressive Complex. Padj: transformed p-value after accounting for multiple testing using Benhamin and Hochberg method by DESeq2.

### DNA-methylation landscape changes under carbon limitation

**Figure 4:**
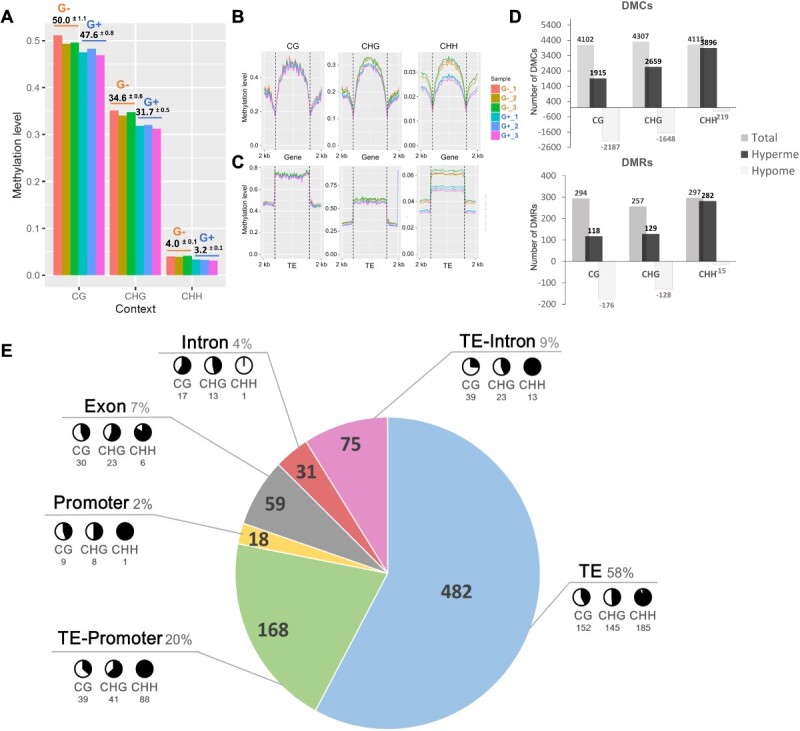
Global DNA-methylation is affected in G- cells. Global methylation level analysis in G− and G+ condition. (A) Bart chart representing the methylation level of individual sample (*y*-axis, 1 = 100%) in the three-cytosine sequence context (*x*-axis). (B, C) Cytosine methylation profiles within and 2 kb up- and downstream of (B) genes coding sequence and (C) of TE. (D) Differentially Methylated Cytosines (DMCs) and Differentially Methylated Regions (DMRs) and their contexts in G− condition compared to G+. Grey bar chart represents the total number of DMC/DMRs identified. Methylation changes are represented in black when methylation increases in G− compared to G+ cells (Hyperme) and in light grey when methylation decreases in G− compared to G+ cells (Hypome). Number of DMCs and DMRs are indicated on the top/bottom of each barchart. (E) Localization of identified DMRs in specific genomic features. Black and white pie charts represent the proportion of hyper-(black) and hypo-(white) methylated of identified DMRs (number underneath) in each sequence context. TE: extragenic transposable elements; TE-intron: intronic transposable elements; TE-promoter; transposable element found within promoter sequence.

To characterize the effects of sugar depletion on DNA-methylation, WGBS was performed on the genomic DNA of G+ and G− cells at D6. A minimum of ~93 million reads were generated for each replicate resulting in a sequencing depth ranging from ~25X to ~74X depending on samples (Table 3). Hierarchical clustering shows that replicates of the same growing conditions cluster together (Fig. S15). The G- cells showed methylation levels of ~50.0%, ~34.6% and ~ 4.0% in the CG, CHG and CHH sequence contexts respectively, which are higher than those of G+ cells, respectively ~47.6%, ~31.7% and ~ 3.2% (Fig. 4A, Tables 4 and 5).

**Table 3:** Whole-genome bisulfite sequencing mapping results and average depth coverage

Sample	Sequence pairs analyzed	Nbr. of paired-end alignments with a unique best hit	Mapping efficiency (%)	Non-uniquely mapped sequences	Average depth coverage
G + _1	130 472 479	82 944 788	63.6	13 213 594	33.97668
G + _2	282 099 730	181 683 529	64.4	29 427 292	74.39895
G + _3	139 655 324	91 667 849	65.6	14 282 757	37.54222
G-_1	96 857 550	59 516 335	61.4	9 877 183	24.38944
G-_2	93 098 553	61 901 086	66.5	10 136 166	25.35674
G-_3	99 426 877	61 857 519	62.2	10 945 369	25.33656

G+: CS cells cultivated in glucose rich medium; G-: CS cells cultivated in glucose poor medium, biological replicates are numbered from 1 to 3.

Analysis of DNA methylation profiles along transcriptional units (ORF +/−2 kb) and Transposable Elements (TE) showed that there is no difference between G- and G+ cells in CG methylation. Methylation is however higher up/downstream and within genes, and at TE in G- than in G+ cells, in both the CHH and CHG contexts, at a lower level in the later context though (Fig. 4B and C).

A total of 12 524 differentially methylated cytosine (DMCs) were identified between G- and G+ conditions with 4102, 4307 and 4115 DMCs in CG, CHG, and CHH contexts respectively (Fig. 4D). The hypo- and hyper-CG-DMCs were similarly abundant, while hyper-DMCs were more abundant than hypo-DMCs in CHG (62%) and represent over 94% in the CHH context (Fig. 4D).

A total of 848 differentially methylated regions (DMRs) were identified between G- and G+ cells (Table S9), with 294 CG-, 257 CHG-, and 297 CHH-DMRs (Fig. 4D). Hypo-DMRs are more abundant than hyper-DMRs in the CG context (176 hypo- and 118 hyper-DMRs), but the converse is found in the CHH context (282 hyper- and 15-hypo-DMRs). A similar number of hypo- and hyper-DMRs is found in the CHG context. We analyzed the distribution of DMRs in different genomic features.

A majority of DMRs (58%, 482 over 848) were localized in TEs, 22% (186 over 848) in promoter (promDMRs), 20% (165 over 848) in genes, distributed between exons (7%, 59 over 848) and introns (13%, 106 over 848). The majority of DMRs in intron (75 over 106) and promoters (168 over 186) are overlapping with TE (Fig. 4E, Table S10). The distribution of hyper- and hypo-DMRs in each sequence context and genomic features is clearly different in TE and genes. The PromDMRs and genic-DMRs occur essentially in the CG and CHG contexts while CHH DMRs are overrepresented in extragenic regions and TEs. Genic-DMRs and promDMRs were mostly hypo-methylated in the CG and CHG context in G- conditions while the majority of TE-DMRs were hyper-methylated in the CHH context.

To assess whether genes involved in metabolism were differentially methylated, genes associated with genic-DMRs (exonic, intronic, TE-intronic) or promDMRs (promDMRs, TE-promDMRs) were used for MapMan analysis. Among the 150 genic-DMRs identified, 48% (72 over 150) were assigned to 17 MapMan metabolic functional categories (Table S11). When considering promDMRs 55% (95 over 173) of them were assigned to 21 MapMan metabolic functional categories including the TCA cycle, amino acid, lipid, protein, nucleic acid and hormone metabolism, stress responses but also transcription regulation and DNA synthesis/repair (Table S12).

### Some DMRs are associated with differentially expressed genes

We determined possible association between DNA methylation and gene expression by looking at the genomic co-localizations between gene- or promDMRs, and DEGs. In total, 17 gene-DMRs were associated with DEGs (DMEGs), and 34 promDMRs were associated with DEGs (promDMEGs; Tables S13 and S14). However, because there is no clear link between gene methylation and expression [25], we only analyzed the 34 promDMEGs for subsequent analysis (Table 6, Table S15).

**Table 4:** Bisulfite sequencing data report

Sample	Total of C’s analyzed	% of methylated C	% of conversion	Methylated cytosines in				% Methylated cytosines in		
D6				CpG	CHG	CHH	Unknown	CpG	CHG	CHH
G + _1	259 133 083	0.567123265	99.43287674	284 782	263 603	920 187	1032	47.6	32.0	3.3
G + _2	546 752 409	0.564586081	99.43541392	608 384	558 261	1 918 117	2126	48.3	32.0	3.2
G + _3	314 771 657	0.601212008	99.39878799	355 313	325 031	1 210 998	1103	47.1	31.4	3.1
G-_1	159 367 452	0.626294759	99.37370524	199 134	180 869	617 520	587	51.2	35.2	4.0
G-_2	168 998 804	0.497389911	99.50261009	179 576	159 608	500 726	673	49.5	34.1	3.9
G-_3	162 348 402	0.614100901	99.38589910	208 515	190 078	597 758	632	49.8	34.8	4.1

G+: CS cells cultivated in glucose rich medium; G−: CS cells cultivated in glucose poor medium, biological replicates are numbered from 1 to 3.

**Table 5:** Global DNA methylation is increased in cells under carbon depletion

% Methylation	CG	CHG	CHH
D6_G+	47.6 ± 0.8	31.7 ± 0.5	3.2 ± 0.1
D6_G-	50.0 ± 1.1	34.6 ± 0.6	4.0 ± 0.1

Methylation average calculated in the symmetrical (CG, CHG) and asymmetrical (CHH) sequence contexts (%), in CS cells cultivated in G+ and G− medium at D6. Error values correspond to CI (*n* = 3).

**Table 6:** Some DEGs are associated with DMRs localized at promoter

N° promDMEG	Gene ID	Chr	Diff. Exp^a^	Diff.meth	Context	Function
1	Vitvi02g01812	chr00	1.35	−0.49	CHG	Unknown
**2**	**Vitvi04g01539**	**chr04**	**2.75**	**−0.28**	**CHG**	**ACT-like protein tyrosine kinase family**
3	Vitvi04g00498	chr04	1.01	−0.27	CG	Myosin heavy chain-related protein
4	Vitvi04g00159	chr04	1.44	−0.25	CHG	electron carriere/heme binding/monooxygenase
5	Vitvi03g00483	chr03	−2.86	0.13	CHH	Carboxylesterase 13
6	Vitvi03g00483	chr03	−2.86	0.13	CHH	Carboxylesterase 13
7	Vitvi04g01733	chr04	−1.39	0.13	CHH	S-adenosyl-L-methionine-dependent methyltransferases superfamily protein
8	Vitvi17g00607	chr17	−1.16	0.13	CHH	L-malate dehydrogenase/oxidoreductase
9	Vitvi08g00969	chr08	−2.41	0.14	CHH	Not in our list
10	Vitvi18g00262	chr18	−1.90	0.14	CHH	IAA-amino acid conjugate hydrolase/ metallopeptidase
**11**	**Vitvi05g02123**	**chr05**	**−2.50**	**0.14**	**CHH**	**Cyclo-DOPA 5-O-glucosyltransferase**
12	Vitvi02g01690	chr00	−2.96	0.15	CHH	Flavin-binding monooxygenase family protein
13	Vitvi03g00755	chr03	−3.27	0.18	CHH	CAP (Cysteine-rich secretory proteins, Antigen 5, and Pathogenesis-related 1 protein) superfamily protein
14	Vitvi18g00430	chr18	−2.98	0.19	CHH	Glucuronosyltransferase/Transcription factor)
15	Vitvi07g00683	chr07	−1.00	0.23	CHH	NTF2 family protein with RNA binding domain
16	Vitvi17g00926	chr17	−1.86	0.25	CHH	Not in our list
17	Vitvi18g02335	chr18	−1.18	0.28	CG	R protein L6
18	Vitvi06g00226	chr06	−1.17	−0.41	CG	Unknown
19	Vitvi18g02508	chr18	−3.37	−0.31	CG	Not assigned
20	Vitvi18g00482	chr18	3.12	0.14	CHH	Purine transmembrane transporter
21	Vitvi02g00805	chr02	1.03	0.15	CHH	Electron carrier/heme binding/monooxygenase
22	Vitvi16g00350	chr16	2.80	0.16	CHH	Integrase-type DNA-binding superfamily protein
23	Vitvi10g00558	chr10	1.53	0.18	CHH	Unknown
24	Vitvi14g00093	chr14	1.07	0.19	CHH	DNA binding/KNOX-like transcription factor
25	Vitvi03g00601	chr03	2.71	0.20	CHH	Binding/catalytic
26	Vitvi11g01472	chr11	1.05	0.24	CHH	Uncharacterized
27	Vitvi14g00118	chr14	1.43	0.24	CHH	Cam-binding protein 60-like G
28	Vitvi10g00924	chr10	1.41	0.25	CHG	No hit
29	Vitvi02g01406	chr02	1.25	0.26	CHH	Thaumatin
30	Vitvi16g01804	chr16	1.18	0.27	CHG	Acyl-CoA oxidases
31	Vitvi08g00716	chr08	1.12	0.29	CG	Receptor like protein 12
32	Vitvi10g02090	chr00	3.10	0.31	CHH	Unknown
33	Vitvi14g01368	chr14	1.07	0.34	CHG	Receptor like protein kinase FERONIA
34	Vitvi02g01753	chr00	2.37	0.37	CHG	Not assigned
^a^Log2FC						

Identified promDMEGs and their associated function; Gene ID: identification based on 12xV2 grapevine genome annotation; Chr: Chromosome; Diff.Exp: Differential expression (Log2FoldChange); Diff.Meth: Difference of methylation between the two conditions. PromDMEGs in bold are non-TE promDMRs.

**Figure 5:**
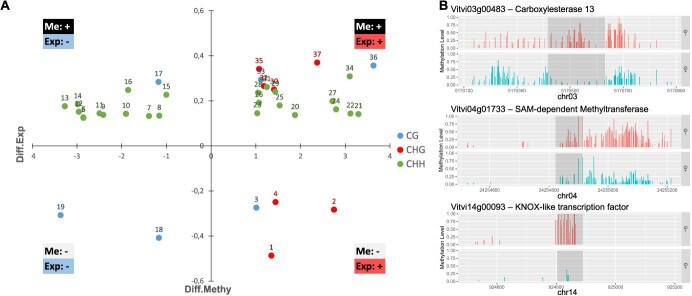
The differential expression of some genes is associated with changes in DNA-methylation at promoters. (A) Graphical representation of the relationship between promoter showing differential methylation and associated changes in gene expression. Each dot represents a DMR found in a DEG promoter region. Position on the graph depends on differential expression value (Log2FC) along the x-axis and differential methylation along the y-axis. Dot numbers refer to those attributed to promDMEGs listed in Table 6. The type of correlation is represented in each corner of the graph. Methylation context is indicated by colors as shown by the legend. (B) Example of methylation patterns in promDMEGs n°6 (*Vitvi03g00483*), n°7 (*Vitvi04g01733*) and n°24 (*Vitvi14g00093*) in G- and G+ cells. Me+/−: methylation changes hyper−/hypomethylated; Exp +/−: gene expression up−/downregulated.

The majority of these promDMEGs (28 over 34) were hyper-methylated, of which 22 (out of 28) were in the CHH context (Fig. 5A, Table 6). The promDMEGs were further classified in four types based on the relationship between their methylation state (Me: +/−) and expression level (Exp: +/−; Fig. 5). Among the 34 PromDMEGs, 17 show a negative correlation between DNA methylation and gene expression levels (Fig. 5, Table 6). They include *Vitvi04g01733* encoding a SAM-dependent methyltransferase, *Vitvi03g00483* encoding a Carboxylesterase (Fig. 5B) and *Vitvi17g00607*, which codes for a l-malate dehydrogenase/oxidoreductase. These genes were hyper-methylated and repressed in sugar-depleted cells (Fig. 5, Table 6). Other genes with hypo-methylated promDMRs were overexpressed. Among them, *Vitvi04g00159* encodes an electron-carrier protein, *Vitvi18g00430,* a 7-deoxylganetin glucosyltransferase with a potential TF activity, and *Vitvi14g00093* a KNOTED1-LIKE HOMEOBOX (KNOX) like transcription factor (Fig. 5B, Ensemblplants, UNIPROTKB ref: AC7Q8). This later gene is orthologous to the *KNAT6* gene known for its role in the regulation of meristematic activity in Arabidopsis [26]. Of note, no DEG involved in the mobilization of carbon resources or participating in the ATG-mediated autophagy pathway showed changes in DNA methylation level in their promoter region.

Overall, results suggest that DNA methylation contribute to the regulation of different genes in response to carbon depletion.

## Discussion

The consequences of carbohydrate depletion on heterotrophic plant tissues were investigated studying CS cells that are strictly dependent on external carbon source. The CS cells were grown in the absence of light with a 10-fold reduction in glucose concentration (2 g/L) as compared to standard conditions (20 g/L). Taking advantage of the efficiency of heterotrophic cell in the control of carbon nutrition [27], this study describes the genome-wide dynamic of DNA methylation and its link with gene expression and metabolic changes in sugar depleted plant cells.

Previous studies in Arabidopsis and rice have shown that the response of cells to carbon limitation is rapid [28] and results in the mobilization of cell carbon resources, such as proteins, lipids, and starch and in the recycling of cellular components by autophagy [23, 27, 29, 30]. Cell viability decreases after 48 h, and cell death is observed after 72 h [23, 30]. Although cell division and death were no evaluated in our study, the CS cell biomass stopped increasing and major metabolic changes, similar to those observed in Arabidopsis and rice cells, occurred 24 h to 48 h after transfer to sugar depleted medium. Total proteins were reduced after 48 hours and the G− cells were depleted in soluble sugar and total amino acid content. Metabolic fluxes required for the resources biosynthesis, including glycolysis and TCA cycle, showed a reduced activity in G− versus G+ cell. Inversely, those involved in resource mobilization including lipid, cell wall polymers and protein breakdown as well as starch mobilization were enhanced (Fig. S7; Table S5). Such a behavior was previously suggested to be an adaptive process enabling the cells to maintain a continuous energy supply under sugar starvation [27].

### Transcriptomic reprogramming is consistent with a major metabolic stress

Changes in glucose availability to plant cells impact the expression of a large number of genes related to stress response, cell wall biogenesis, cellular metabolism, cell signaling, and transcription factors [31]. While end products of PS such as sucrose and glucose repress photosynthetic genes [32], carbon depletion results in the induction of genes involved in PS and in nutrient mobilization from starch, sucrose, and lipids, as alternative carbon source (Contento, Kim and Bassham, 2004; [30]). Consistently, in our study, CS cells in G− conditions were characterized by a very strong upregulation of genes involved in PS, particularly in the light reactions (Fig. 3B, Figs S10, S12 and S13), associated with the downregulation of those involved in cell cycle, biomass production including cell wall synthesis, glycolysis, nucleotides formation and the TCA cycle (Fig. 3A). In contrast, genes contributing to cellular and metabolic processes associated with carbon mobilization such as autophagy, glyoxylate cycle, lipid, starch, and sucrose breakdown pathways were upregulated (Fig. 3B, Figs S10, Fig. S11, Fig. S12, Table S8).

These results are consistent with those obtained in sucrose-starved non-photosynthetic cells of Arabidopsis (Contento, Kim and Bassham, 2004) and demonstrate that CS cells present typical effects on their metabolism, gene expression patterns, and phenotypes of cells under severe carbon depletion.

### Cells under carbon depletion present changes in DNA methylation levels and distribution

One important aspect of our work was to investigate whether carbon depletion could impact on the synthesis of SAM, the universal methyl donor, which in turn may affect methylation processes in plant cells, including DNA methylation. Arabidopsis plants mutated in 1C key regulatory genes such as Methylenetetrahydrofolate-dehydrogenase or MET-adenosyltransferase (respectively, *mthfd1–*1 and *mat4*) show altered DNA and/or histone methylation profiles [33, 34]. In our study, at D6, both transcriptomic results and flux analyses demonstrated, respectively, the downregulation of genes involved in the methylene-THF cycle, and a reduction of fluxes through the THF pathway in G− condition (Figs 2 and 3, Fig. S12, Table S6). However, fluxes toward DHF synthesis were only slow down at D6 (Fig. 2; Table S6), whereas those necessary for SAM synthesis were totally stopped, suggesting that the synthesis of SAM is not prioritized over other metabolites of the 1C metabolism. Hence, sugar depletion may result in limited availability of SAM and as a consequence of methylation activities.

### Carbon limitation induces global DNA-methylation changes

Plant cell DNA-methylation landscape is modified in response to environmental changes in plants or when plants are facing nutrient starvation as for example phosphate limitation [35–37]. The present work is showing that global cytosine methylation is higher in G− than in G+ condition with a 4%, 8%, and 20% increase in the CG, CHG and CHH context respectively (Fig. 4A; Tables 3 and 4). Consistently, the DNA-methylation level within genes and in 2 kb regions up/downstream of genes was higher in the CHG and CHH contexts in G− as compared to G+ conditions whereas no significant difference was found in the CG context (Fig. 4B). A similar observation is made for TE, as hypo- and hyper-DMCs in the CG context were equally abundant, while CHG hyper-DMCs were more abundant than CHG hypo-DMCs and almost all CHH DMCs were hyper- in G− cells (Fig. 4C, D).

Although unexpected, these results are consistent with recent studies showing that the efficiency and timing of DNA-methylation maintenance differs between the C sequence context during cell division [38]. The CG methylation is transiently loss during the S phase and rapidly reestablished at the G2 phase. In contrast, maintenance of CHG methylation is delayed resulting in hemi-methylated sites that are reestablished when cell division stops, which leads to a transient reduction of the CHG DNA-methylation. The strongest effect is observed on CHH methylation, which is depleted in actively dividing cells in culture, probably as a result of the loss of small RNA during the cell cycle [38]. In our study, G+ cells were actively growing from D4 to D8 (Fig. 1B), when G− cells did not, as shown by the absence of FW increase (Fig. 1A). This is most likely due to an arrest of cell division of G− cells, considering the downregulation of cell cycle-related genes (Fig. 3A). Thus, the CHH and CHG hypo-methylations observed in G+ versus G− cells might in part reflect the difference in cell division activity between the two conditions of our study. However, we cannot rule out that the dynamic of CHH DNA methylation is also a direct consequence of the metabolic stress generated by carbon depletion. In grapevine, and in other plants, CHH methylation is highly dynamic in plants under stresses [11, 39], an observation associated with the regulation of *VvAGO*, *VvDCL*, and *VvRDR* gene families under abiotic stresses in grapevine [40]. We have controlled the expression of *DNMT* genes and of genes involved in DNA-methylation regulation. The *VvMET1* gene was similarly expressed in both conditions, while *VvDDM1*, all *VvCMT* and *VvDML3* were down-regulated in G− cells. Hence there is no clear correlation between *DNMT* gene expression and DNA-methylation changes, an observation already performed in plants [36]. As small RNAs are depleted in dividing cells [38] our analyses also focused on genes encoding putative ARGONAUTE (AGO), RNA-directed-RNA-Polymerase (RDR) and DICER-LIKE (DCL) proteins, which are known to participate in the RdDM pathway by controlling siRNA synthesis [41]. The *VvAGO10a* and *VvAGO11* were under-expressed in G− versus G+ cells, in contrast to *VvAGO5, VvAGO2a*, *VvRDR1b*, and *VvDCL2* which were over-expressed (Table 2). This complex expression patterns most likely reflects a dynamic regulation of the RdDM pathway in G- cells. Whether this could contribute to the hyper-methylation observed in the CHH and CHG contexts in G− cells remains to be demonstrated.

### The majority of DMRs are localized at transposable elements

DMRs are usually considered as functional regions associated with the control of gene expression and transposon activity. We have identified 848 DMRs that were equally distributed between CG, CHG, and CHH sequence contexts (Fig. 4D). Consistent with the meta-analysis of DNA methylation at genes and TEs and with DMCs distribution, hypo- and hyper-DMRs in the CG and CHG context were similarly abundant, unlike CHH-DMRs presented an important bias toward hyper-DMRs in G− versus G+ cells (Fig. 4D).

Most DNA methylation changes irrespective of the C sequence contexts were associated with TEs (Fig. 4E). The TE associated DMRs also showed an overrepresentation of hyper-DMRs in the CHH context, as compared to CG and CHG context which were equally distributed between hyper- and hypo-DMRs (Fig. 4E). In contrast, those located at promoters and gene bodies and not associated with TEs were mainly found in the CG and CHG context and did not show any specific trend toward hyper- or hypo-methylation. This suggests that depending on their genomic features, DMRs triggered by difference in carbon availability in CS cells are generated by distinct mechanisms.

Hyper-methylated CHH at TEs have already been reported in various plants subjected to abiotic stresses, as for example, in maize [42], as well as in tomato plants under phosphate starvation [36]. As TEs are targeted by repressive epigenetic marks as a preservation mechanism for genome integrity [43], this response has been associated with the activity of the RdDM pathway to transposons repression in accessible chromatin environments [44]. In our experiments, although we had not analyzed the small RNAs populations, it is likely that siRNAs are depleted in G+ conditions as cells are actively dividing. Therefore, hypo-methylated DMRs at TEs in G+ conditions might also be due to a loss of methylation in G+.

Overall, results showed that sugar limitation results in important changes in DNA methylation patterns in all sequence contexts mostly located at TEs. Furthermore, the high number of hyper-CHH argues in favor of the involvement of the RdDM pathway in response to sugar availability, either leading to a decrease in methylation in actively dividing cells [38] or strengthening it in cells that present a nutritional stress, or a combination of both.

### Differential methylation may have an impact on gene expression in cells under carbon depletion

Among the 848 DMRs identified, 165 (19%) were located in genes and 186 (22%) in their promoter region (−2 kb upstream the TSS; Fig. 4E). Among them, 90 and 18 were not associated with TEs. Interestingly, intragenic-DMRs were preferentially CG- (86 over 165) and CHG-DMRs (59 over 165) with very few CHH-DMRs (20 over 165). All except one intragenic CHH-DMRs were hyper-methylated and associated with TE. The Gene-CG-DMRs were mostly hyper-methylated (53 over 83) in G− cells, suggesting that this differential methylation might be directly linked to a response to carbon depletion, as was observed for promDMRs not associated with TE. The DMRs located in genes and promoters were enriched in genes related to the central and secondary metabolism, along with those involved in stress response, photosynthesis, chromatin structure, protein PTMs, and transcription regulation (Tables 6, S10, and S11).

Among the 5607 identified DEGs, 34 present DMRs located in their promoter regions, among which 17 show a negative correlation between methylation and gene expression. These 17 promDMEGs include genes coding for putative transcription factors such as a Class 1 KNOX gene, enzymes involved in the central metabolic pathways including a malate dehydrogenase, and an S-adenosyl-methionine-dependent methyltransferase among others (Fig. 5B, Table 6). This observation strongly suggests that DNA-methylation is involved in the control of gene expression as an integrated part of the cell metabolic and molecular responses to carbon depletion. Similarly, differentially expressed genes related to phosphate starvation regulation in tomato plants under low phosphate conditions display DNA-methylation changes [36]. Hence, DNA-methylation dynamic likely represents a general strategy of plant cells to deal with nutritional stresses, such as carbon depletion.

However, additional regulatory processes, including other epigenetic mechanisms may also be at work. Consistently, many DEGs participating in cellular processes involved in the responses of grapevine cells to carbon starvation were not associated with changes in DNA methylation levels in their promoter regions, including DEGs involved in autophagy, protein or lipid mobilization. However, as for DNA methylation, HPTMs also rely on the availability of metabolic precursors and cofactors. Several DEGs encoding HMTs were identified and shown to be downregulated in CS cells under glucose depletion (Table 2), consistent with a limitation of most methylation processes associated with epigenetic modifications in G− cells. Moreover, inhibition of fluxes involved in the TCA cycle was observed in G− cells, that could result in change in histone methylation patterns as observed in mammalians [45].

Overall, results showed that epigenetic changes under carbon shortage are complex and may rely not only on DNA methylation but also involve histone post-translational modifications. The differential expression of histone modifiers in G− versus G+ cells suggests that the distribution of histone marks may also be affected under sugar depletion. Recent reports have shown that autophagy, a major process involved in cell response to carbon starvation is associated with changes in histone mark distribution including the acetylation of Lys16 of histone 4 (H4K16ac) and dimethylation of H3K9 (H3K9me2) at genes involved in the autophagy pathway in mammals [46, 47]. Further analysis is now needed to assess the changes in HPTMs distribution induced under carbon depletion and their relation with gene expression in plant cells.

In summary, our study provides a comprehensive genome-wide analysis of DNA-methylation changes and their relationship with gene expression to orchestrate metabolic adjustment in the response of plant cells to sugar depletion. In particular, we have demonstrated an important DNA methylation remodeling under carbon depletion that can be associated with changes in the expression of genes encoding enzymes involved in the cell metabolism but also transcription factors that in turn may coordinate the response of cells to carbon depletion.

## Materials and methods

### Cell culture

Grape cell suspensions of CS Berries (CSB, [48] initiated from fruit tissues were cultured in 50 mL of liquid Murashige and Skoog medium (MS, Sygma M0221) supplemented with vitamins (0.025 g/L of *myo-*inositol, 0.25 mg/L of nicotinic acid, calcium pantothenate, HCL pyridoxine, HCL thiamine and 0.0025 mg/L of d(+)-Biotin), 0.6 mg/L of benzylaminopurine (BAP), 2.3 mg/L of naphthalene acetic acid (NAA), 0.25 g/L of casein enzymatic hydrolysate (Sigma C0626), and 20 g/L of glucose in 250 mL Erlenmeyer at 23°C in darkness on an orbital shaker (120 rpm). Cells were sub-cultured weekly by transferring 10 mL of the cell suspension to 40 mL of fresh medium, in individual vials for each time point and condition. Sample collection was performed every day from the day of inoculation (D0) to ten days after (D10) at the same hour. Cell growth was estimated by measuring the cell fresh weight (FW) in 30 ml culture after removal of the culture medium by filtration. Cells and culture medium were subsequently frozen in liquid nitrogen, ground to a fine powder in a Retsch Mixer Mill MM 400 (Fischer Scientific), and stored at −80°C until processed.

For sugar depletion experiments, cells cultured during four days in standard condition (SD) were transferred to either a glucose rich (G+: 110 mM i.e*.*20 g/L) or glucose poor (G-: 11 mM i.e., 2 g/L) medium (Fig. S1B) and grown for 6 additional days. The cell water content was evaluated using ~140 to 200 mg FW of frozen (maintained at −80°C) grounded cells, by comparing the cell mass before and after lyophilization.

### Metabolic analysis

Glucose and fructose concentrations in culture medium were measured enzymatically with an automated absorbance microplate reader (Elx800UV, Biotek Instruments Inc., Winooski, VT, USA) using the glucose/fructose kit from BioSenTec (Toulouse, France) according to manufacturer’s instructions. The results were presented as ‘extracellular glucose’ concentration. Extractions for both targeted and untargeted metabolite analyses were performed in quadruplicate for each sample of frozen cell powder.

Extraction was performed as described in [49] at the *Bordeaux Metabolome Facility* (https://metabolome.u-bordeaux.fr/en/, Villenave d’Ornon, France) with the following modifications: [42] 20 mg of fresh frozen powder (FW) of each replicate sample were used for extraction, [35] ~10 mg of polyvinylpolypyrrolidone (PVPP) were added in each tube prior to extraction to precipitate polyphenols.

Analyses of soluble sugars (glucose, fructose, sucrose), starch, malate, citrate, total soluble proteins, total amino acids were conducted at *Bordeaux Metabolome Facility*. Measurements were based on coupled enzyme assays as described previously in [50], except for total soluble proteins measured by Bradford assay [51].

Extraction of NAD+, NADH, NADP+, and NADPH was performed from 20 mg of fresh frozen grapevine cell powder (FW) with the addition of ~10 mg of PVPP prior to extraction and quantified as described by [52, 53]. Quantification was performed in technical duplicate using four independent biological replicates.

Analysis of 19 amino acids (excluding tryptophane and cysteine) was performed essentially as described by [54]. Briefly, extracts were derived with 6-aminoquinolyl-*N*-hydroxysuccinimidyl carbamate (AccQ-Tag derivatization reagent, Waters, Milford, MA, USA) and analysed using an UltiMate 3000 UHPLC with an FLD-3000 Fluorescence Detector (Thermo Electron SAS, Waltham, MA, USA). Free amino acids’ separation was achieved with an AccQ-Tag Ultra column, 2.1 × 100 mm, 1.7 μm (Waters, Milford, MA, USA) at 37°C with elution at 0.5 mL/min (sodium acetate buffer, 140 mM at pH 5.7; acetonitrile; water) and detection was performed at 395 nm with an excitation wavelength at 150 nm.

Analysis of cell wall biomass was performed on pellets resulting from the metabolite extraction described above following the *Bordeaux Metabolome* platform protocol. Briefly, after metabolite extraction, pellets were separated from supernatant and resuspended in 250 μL of NaOH 0.5 M at 95°C for 20 min. Supernatant was removed after centrifugation, the pellet was washed twice by addition of 250 μL of water and centrifuged at 2500 rpm for 10 min. After overnight lyophilization, cell wall content was estimated by calculating the difference of tube weight before and after discarding the pellet.

Untargeted metabolic analysis were performed as described in [55, 56], using four independent biological replicates (*n* = 4). The analytical sequence contained 10 Quality Control (QC) samples, prepared by mixing 20 μl of each sample, to correct the signal drift during the analytical batch and to the calculation of coefficients of variation for each metabolomic feature optimized parameters (Supplemental Material 1). Putative annotation of differentially expressed metabolites was performed by screening the MSDIAL online library (MSMS-Public-Neg-VS15, http://prime.psc.riken.jp/compms/msdial/main.html#MSP; [57]) using the precise *m/z* of the parent ion and MS^2^ fragmentation spectra.. After data-cleaning (blank check, S/N > 10, CV(QC) < 30%), the dataset was normalized (median normalization, cube-root transformation and Pareto scaling) using MetaboAnalyst (v 5.0; [58] prior to multivariate statistical analyses.

### Flux-balance model

The flux-balance model of heterotrophic plant central metabolism used here was initially described in [59] and improved (details given in [60] to better describe all the reactions involving energy, metabolic compounds for biomass synthesis (proteins, cell wall, lipids, nucleotides, amino acids) and metabolic precursors for secondary metabolism. Details are provided in supplementary information.

### Software

Stoichiometric model (in *sbml* format, supplementary material) and mathematical problems were implemented using MATLAB (Mathworks R2018, Natick, MA, USA), solver *quadprog* with interior-point-convex algorithm for the minimization.

### Molecular characterisation

#### Nucleic acid extraction

Both DNA and RNA were both extracted from the same sample (~200 mg cells of fresh frozen powder, FW) of independent biological replicates (*n* = 3) of each conditions (D4, D6 G+, D6 G−) according to [61].

#### Bioinformatic analysis of RNAseq data

High-throughput sequencing of RNA samples were performed using DNBSEQ Sequencing technology (pair-end, 150 bp) service provided by BGI-Genomics platform (http://bgi.com). We generated 9 cDNA libraries corresponding to three biological replicates for each group (D6 G+ and D6 G-). Raw reads were trimmed using Trimmomatic v0.38 in PE mode [62]. The sequence alignment files were generated by STAR (version 2.5.1b, [63]. To generate the raw gene counts, we used the *featureCounts* function of the Rsubread package [64] which assigns mapped sequencing reads to genomic features based on the grapevine reference genome assembly of PN40024 12X.2 (https://urgi.versailles.inra.fr/Species/Vitis/Data-Sequences/Genome-sequences). We used the DESeq2 package to identify differentially expressed genes [65]. After selection of genes presenting a count per million reads (CPM) >10, differentially expressed genes (DEGs) were identified using a False Discovery Rate (FDR)-adjusted p-value threshold <0.05. In addition, only DEGs with a log2 fold change >1.0 were selected.

#### Bioinformatic analysis of WGBS data

Whole Genome Bisulfite Sequencing of DNA samples were performed using DNBSEQ-sequencing (pair-end, 100 bp) technology provided by BGI-Genomics. Reads obtained from the WGBS approach were first trimmed with TrimGalore! (Version v0.4.5). First, we assessed the bisulfite conversion rate using the unmethylated grapevine chloroplast genome with Bismark tool (version v0.20.0, [66]. Cleaned reads were then aligned onto the grapevine reference genome PN40024 (12Xv2) using Bismark (version v0.20.0) allowing 5 mismatches. Reads with multiple alignment were discarded. PCR duplicates were also removed using the *deduplicate_bismark* tool. The methylation state of each cytosine was calculated, for the three contexts CG, CHG and CHH. We then used the DSS R package (dispersion shrinkage for sequencing data - version 2.39.0, [67] to identify DMRs based on a Wald test procedure and accounting for both biological variations among replicates and sequencing depths. First, differential methylation statistical tests were performed at each C locus by calling the DSS *DMLtest* function with smoothing the methylation levels using a simple moving average algorithm. Then, differentially methylated loci were retained when the difference in mean methylation levels was >0.1 for CG or CHG contexts and > 0.07 for CHH context with a posterior probability >0.9999. DMRs were then identified using the DSS callDMR function with standard parameters. To define hypo- or hyper-DMRs, we applied a cut-off of at least a 10%, 25%, and 25% change in methylation ratio for CHHs, CHGs, and CGs, respectively.

#### MapMan analysis

All the gene models were automatically categorized according to the MapMan ontology (x3.6) with Mercator tool [68] and MapMan standalone software v3.5.1 [69] was used to explore the data.

### Statistical analysis

Untargeted metabolic profiling data were checked for statistical significance by ANOVA for global variation. Significant differentially accumulated metabolites among conditions considered (Fisher’s *P* < 0.01) were determined and analyzed using MetaboAnalyst 5.0 online software (https://www.metaboanalyst.ca/docs/Format.xhtml).

For all other analyses, mean and confidence interval values (CI) were calculated with R software (v4.1.1). Data were checked for normality using Shapiro–Wilk test, and further checked for significance by performing parametric (*t*-test) or nonparametric (Wilcox–Mann–Whitney test) depending on their normality status.

## Supplementary Material

Web_Material_uhae277

## Data Availability

The data for this study have been deposited in the European Nucleotide Archive (ENA) at EMBL-EBI under accession number PRJEB72068 (https://www.ebi.ac.uk/ena/browser/view/PRJEB72068).
