# Supplementary material for: Grapevine cell response to carbon deficiency requires transcriptome and methylome reprogramming
Source: Hortic Res. 2024 Sep 28;12(1):uhae277. doi: 10.1093/hr/uhae277 (PMC11750959; doi:10.1093/hr/uhae277)
Supplement: Web_Material_uhae277 [file web_material_uhae277.zip › Cell sugar starvation_Supplementary Informations.docx]

**Supplementary Informations**

**SI Results**

**Untargeted metabolism analysis (Figure 1, Table 1)**

At D8, glucose is no longer detectable in G+ medium, consistent with the end of cell exponential growth (**Figure 1A**). At that times, the number of differentially accumulated metabolites between G+ and G- reach 433, before decreasing from 213 to 132 at D9 and D1, respectively (**Table 1**). This suggest that G+ undergoes they own transition from active growth to a lagging phase due to progressive carbon depletion in the medium (**Figure 1A**). This is further supported by the reduction of differentially accumulated metabolites observed between G+ and G-, and consistent with G+ drift observed on the PCA from D8 to D10 (**Figure 1B**). The number of differentially accumulating metabolites reached 433 at D8 before decreasing from 213 to 132 at D9 and D10, respectively (**Table 1**). To avoid any observation bias due to naturally occurring carbon depletion in G+ cells at D8, we focused on comparing G+ and G- metabolomics profiles observed from D4 to D7.

**Medium osmolarity evolution (Figure S2)**

Rapid evolution of extracellular glucose contents impacts global medium osmolarity. Glucose concentration in the medium decreased rapidly in G+ cells **(Fig. S2A)**, at a higher rate as compared to SD (D0 to D4). In the SD condition, extracellular glucose concentration decreased from 13.3 (± 0.97) g/L at D0 to 11.57 (± 0.26) g/L at D1, and from 14.46 (± 0.94) g/L at D4 to 10.16 (± 0.50) g/L at D5. This difference most likely reflect the higher number of cells present at D4 after subculturing the cells in G+ medium (156.7 ± 10.2 gFW/L) as compared to the cell amount at D0 (66.8 ± 7.0 gFW/L), thus increasing extracellular glucose consumption rate. At D6, the glucose uptake rate was higher in G+ than in G- (5.44g/L/day and 2g/L/day respectively). In G- condition, glucose was no longer detectable at D5 in contrast to G+ conditions whereas glucose was detectable up to D7. At D6, the glucose uptake rate was the highest (5.44 g/L/day) in G+ condition and was null in G- condition. This resulted in different Osmotic Pressure (OP) between conditions, that could be evaluated based on the measured glucose concentration in the medium with a maximum of 110 mOs delta between G+ and G- medium observed at D4 **(Fig.S2B)**. This difference was however transient and no more detectable from day 7. As a conclusion, limiting carbon availability to cells leads to a dramatic and rapid reduction of cell growth. Regarding glucose uptake rate, D6 appears as the most contrasted conditions to study grapevine cell response to carbon deficiency.

**Targeted metabolites analysis (Figure S5**)

In pre-cultured cells (D0 to D4), the glucose and fructose cell content did not vary significantly (**Fig.S5 B,C**), in contrast to sucrose that increases rapidly from D0 (3.13 ± 2.6 µg.eq.glucose/gFW) to peak at D2 (51.76 ± 8.06 µg.eq.glucose/gFW), before decreasing to reach 19.39 ± 1.94 µg.eq.glucose/gFW at D4 (**Fig.S5 A**). Although the accumulation of total amino acids gradually decreased from D0 to D4, individual amino acids presented various accumulation profiles (**Fig.S5**). After the transfer of cells to G- condition all soluble sugars rapidly dropped from D4 to reach levels below detection at D6 (**Fig.S5 A-C**). In contrast, in cells transferred in G+ conditions, intracellular glucose content rapidly increased to 61.6 ±12.8 µg /gFW at D5 and then decreased following a biphasic kinetic to reach 3.2 ± 2.6 µg/gFW at D10. A different profile is observed for fructose, which presents a weak and transient increase at D5 followed by a progressive decrease to reach a plateau at D6 that is maintained up to D10 at a level similar to SD conditions (**Fig.S5 C**). Like glucose, the cell sucrose content increased over 2-fold from D4 to D5 in G+ conditions before decreasing to levels below detection at D8 (**Fig.S5 A, B**). To the exception of a transient increase in malate content at D5 in G+ cells (**Fig.S5 E**), total protein and malate abundance showed little difference in accumulation profiles between G+ versus G- conditions (**Fig.S5 E,F**). In contrast, the abundance in total amino acid contents decreased significantly from D6 on, although amino acids presented five different profiles of accumulation (**Fig.S5 G, Fig.S6**). Inversely, citrate started to accumulate since D5n G- condition (**Fig.S5 D**).

The NAD(P)H/NAD(P) ratio (reduced/ oxidized forms) a good indicator of the redox state of cells (Xiao *et al.*, 2018). Both NAD(H) and NADP(H) were measured, and their reduced/oxidized ratio calculated (Fig.S5 H, I). The NADPH/NADP and NADH/NAD ratio did not show significant variations during the preculture (D0 to D4). However, after subculturing, the NADPH/NADP ratio was highly variable in both conditions from D5 to D7 **(Fig.S5 H**). At later times, it increased in G+ cells to reach 1.18 ± 0.58 at D10, twice more than the value of 0.58 ± 0.22 calculated in G- conditions. The NADH/NAD ratio showed a significant increase, from D6 (0.07 ± 0.04) to D10 (0.18 ± 0.02) in G+ condition and an important decrease in G- condition, to reach a final value below 0.1 (**Fig.S5 I**). Hence glucose limitation impacts the redox balance in grapevine cells, with a progressive decrease of the oxidized relative to reduced form of both NAD and NADP.

**Amino acids accumulation (Figure S6)**

According to their accumulation profiles, we identified five types of variations in amino acid abundance. (1) Methionine, glutamine, lysine, GABA, serine and histidine did not show major differences in accumulation pattern between G+ and G- conditions from D4 to D10 although glutamine contents in G+ were slightly higher than in G- cells. The second group (2) gather asparagine, arginine and threonine, showing contrasted profiles between G+ and G- conditions. Indeed while no accumulation variation was detected in G+ condition, G- cells showed an exponential increase of these compounds from D4 to D10. By contrast, the third type of accumulation profile (3) concerns alanine, proline, and glycine contents, characterized by a progressive decrease from D4 to D10 in G- cells, while G+ cells displayed a strong increase from D4 to D7 followed either by a plateau, or a slight decrease from D8 to D10. The fourth group (4) is composed by phenylalanine, tyrosine, and aspartate. Except for aspartate, these compounds showed similar accumulation profiles in G+ and G- condition at D4 and D5. In G+ cells, the three amino acids were progressively accumulated from D6 to D10. In G- cells, these compounds are strongly accumulated and form peak at D6 (aspartate) or D8 (phenylalanine and tyrosine) followed by a decrease until D10. The last group (5) include valine, leucine and isoleucine characterize by a decrease from D4 to D5 followed by exponential accumulation in G+ cells from D6 to D10. All three amino acids are rapidly accumulated in G- cells to reach a plateau at D6, followed by a progressive decrease from D8 to D10. Glutamate was not assigned to a group according to its singular behavior. In G+ cells, glutamate contents increased to a peak (D5), and progressively decreased to stabilize at D8 at value observed at D4. In G- cells, equivalent and sequential increase and decrease are observed between D4 and D6 to stabilize at the same value as the one observed in G+ condition.

**Metabolic fluxes analysis (Figure S7)**

Calculating flux analysis allows revealing carbon inputs, such as glucose, to condition end-products as key drivers of metabolic behavior (Sweetlove, Obata and Fernie, 2014; Clark *et al.*, 2020). Primary metabolism can be described by medium-scale models reconstructed from biochemical and bibliographic knowledge (Colombié *et al.*, 2015), including specialized or secondary metabolic pathways (Lacrampe *et al.*, 2024). Because of remaining glucose in the medium at D4 in G+ condition and of limiting glucose after D7 in both conditions, we focused on D6 to evaluate the consequences of change in carbon availability on the metabolic fluxes and to integrate results with those of molecular analyses. We were mainly interested in the changes of fluxes as a way to evaluate metabolism reprogramming under carbon starvation. The comparison of fluxes (**Table S5**) shows that the main changes in external fluxes (used as constraints in the metabolic model) in G- is an increase of fluxes toward amino acids synthesized from oxaloacetate (OAA) and a decrease of those of cell wall synthesis and hexoses storage. Most of the internal fluxes calculated in G- were lower than the ones calculated in G+ at D6 including those of glycolysis, oxidative phosphate pathway, and cell wall biosynthesis, and to a lower extend those of respiration, nitrate assimilation, and of the TCA cycle. Interestingly, some fluxes were increased under carbon limitation. They include fluxes involved in the mobilization of stored compounds, such as cell wall, protein, as well as the accumulation of lipids, and of some amino acids. This led to an increase of internal fluxes mainly through fructokinase, malate dehydrogenase, malic enzyme, aminotransferases (aspartate and alanine) required to generate the precursors to support the synthesis and accumulation of amino acids and organic acids and the redox fluxes towards ascorbate and glutathione accumulation. Consistent with the requirement of organic acid and amino acid storage, a higher flux partition is observed towards the anaplerotic pathway (*Vpepc/Vpepc+Vpk*) ratio higher in G- (0.371) than in G+ (0.293) at D5). These flux results show a clear metabolic stress for cells under carbon limitation.

**SI Methods**

*Flux balance model*

Briefly, this model (**Tables S1-S2, Supplementary Material 2**) took into account the main pathways, such as glycolysis, tricarboxylic acid (TCA) cycle, oxidative pentose phosphate pathway, sucrose catabolism, etc, using glucose as carbon source (*Vglc_up*), nitrate as nitrogen source (*Vno3_up)* and glutamine as both (*Vgln_up*). All the cofactors were defined as internal metabolites, which means that they were balanced, thus constraining the metabolic network not only through the carbon and nitrogen balance but also through the redox and energy status.

The model described the cell metabolism through a set of 302 reactions involving 177 internal metabolites. At steady state, the mass balance equation was expressed by

dX/dt=NV=0 (1)

X is the vector of internal metabolites, V the flux vector composed of n reactions of the network, and N the stoichiometry matrix. To solve the system, constraints were applied on each flux. First, thermodynamic properties were used to constrain fluxes from reversibility to irreversibility. Thus, among the internal reactions of the metabolic network, lower bounds of irreversible reactions were set to zero. The most important constraints were the external fluxes, also called exchange fluxes. They were calculated from the accumulated metabolites and biomass components, covering an average of 75% of the dry biomass under control and carbon-limiting conditions (**Table S3**). Similarly, the flux of glucose uptake rate was calculated from extracellular glucose concentration. To achieve this, the best polynomial fitting (of experimental data expressed in mmol/L) was searched and the corresponding flux was calculated by derivative (**Table S4**). Finally, flux minimization, which leads to a unique solution (Holzhütter, 2004), was used as the objective function to solve the system, and generate flux maps each day before and after carbon limitation (**Table S5**).

**SI Supplementary Figures**


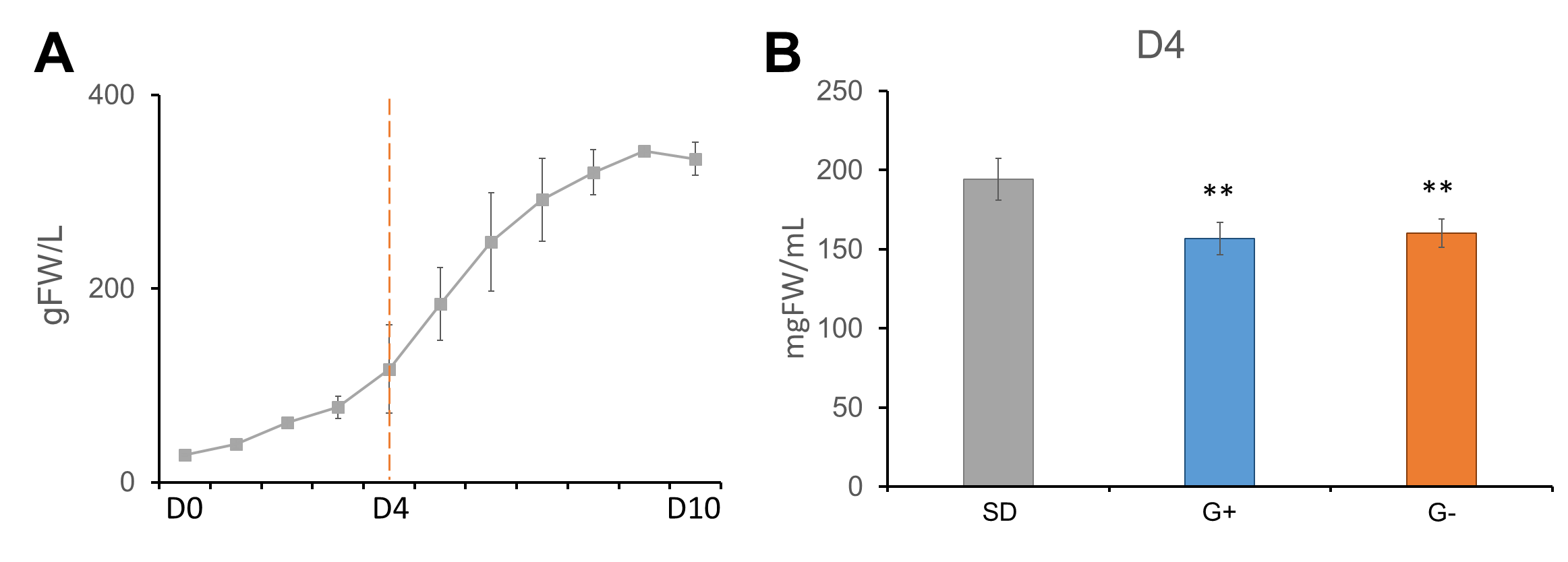


**Figure S1: (A) Cell growth curve of Cabernet Sauvignon (CS) cells in standard condition (SD) (n=2) (B) Fresh weight (FW) after medium change between Standard (SD) and G+/- conditions. Cell concentration presented in mgFW/mL was estimated before (SD) and after the transfert in a new medium (G+ and G-). To achieve this, cells were decanted for a few minutes, and the buffer removed by pipetting. Cells were washed three times with either G+ or G- fresh medium, depending on the conditions, and re-suspended in the appropriate medium. **: pval ≤ 0.01**


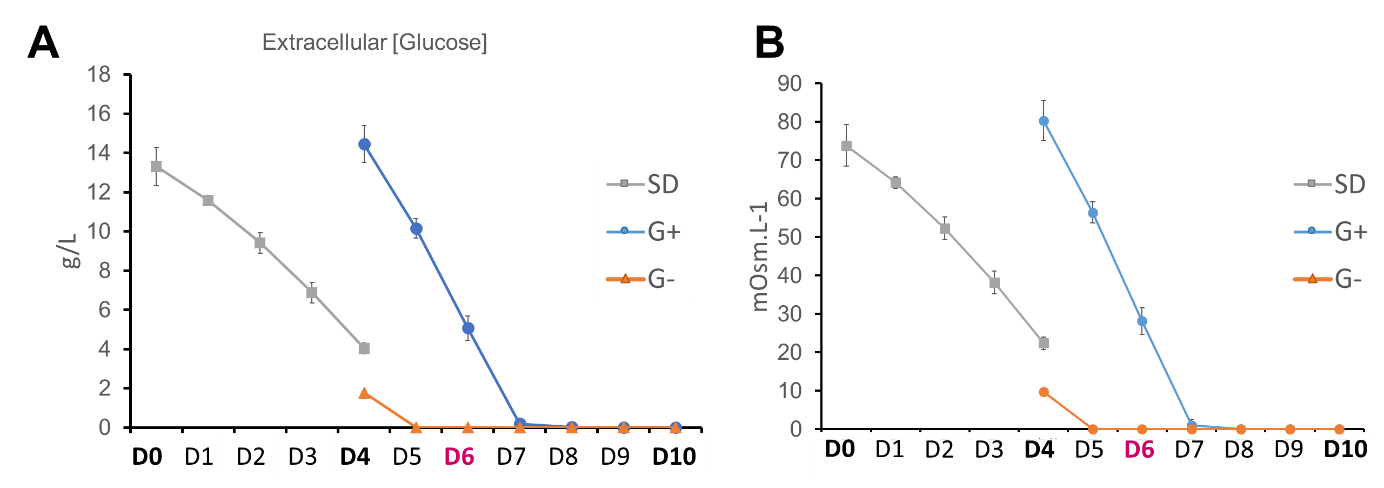


**Figure S2: Medium glucose concentration (A) and osmolarity (B) evolution of SD, G+ and G- conditions.**


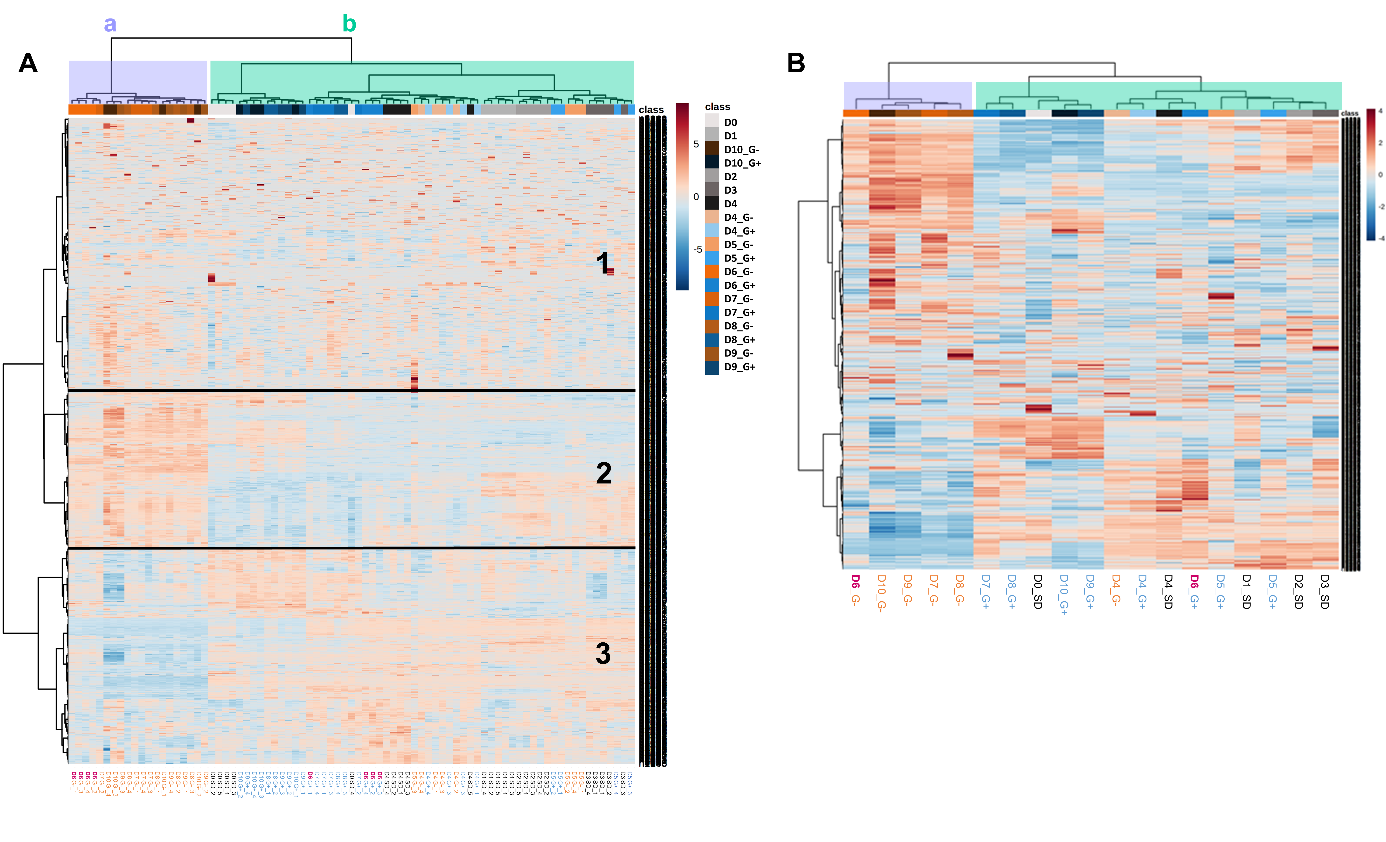


**Figure S3: Heatmap representation of samples metabolic profiling based on the enrichment value of the 1719 metabolic signature (normalized per row) detected using LCMS analysis, and clustered using Pearson’s correlation. (A) Representation of metabolic profile of each sample replicates ate each time point (n ≥ 4) and (B) averaged metabolic profile per day and condition.** The heatmap presented in (A) was separated in three horizontal parts (1, 2, 3) according to the pattern of metabolic features of accumulation within the samples. Part 1 groups the metabolites that are not differentially accumulated depending on the samples. The second group (2) represents metabolites that are more accumulated in D6 to D10 G- samples, and the last one (3) is composed of metabolites less detected in D6 to D10 G- samples, showing the clear differentiation between G+ and G- condition from D6, the latter illustrated by a vertical separation. Samples separated in two cluster (a, light purple; b, light green) based on their metabolic features.


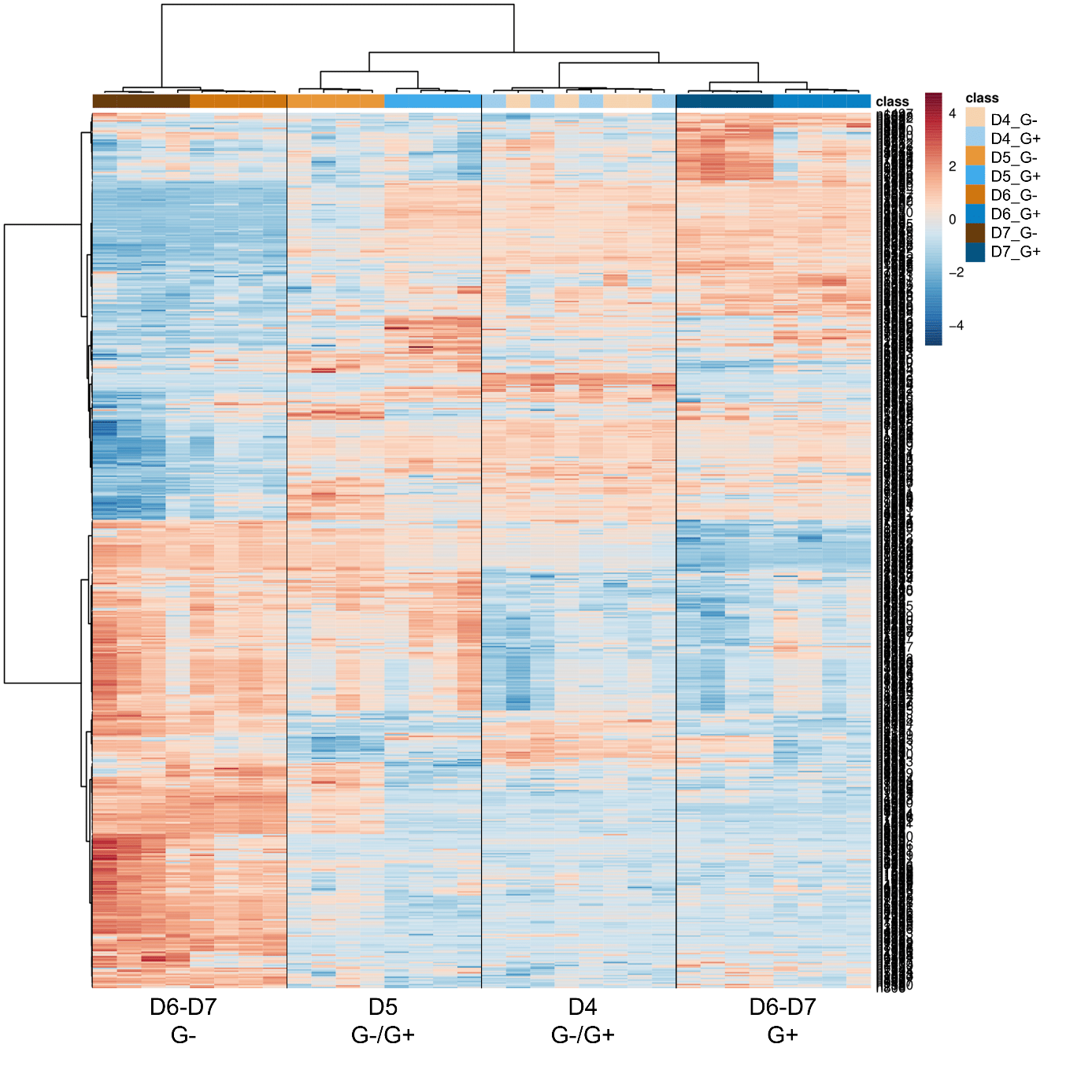


**Figure S4: Heatmap representation of samples profiles from D4 to D7 in G+ and G- conditions based on the 596 most significantly differently accumulated metabolic features across all conditions (p < 0.01)**


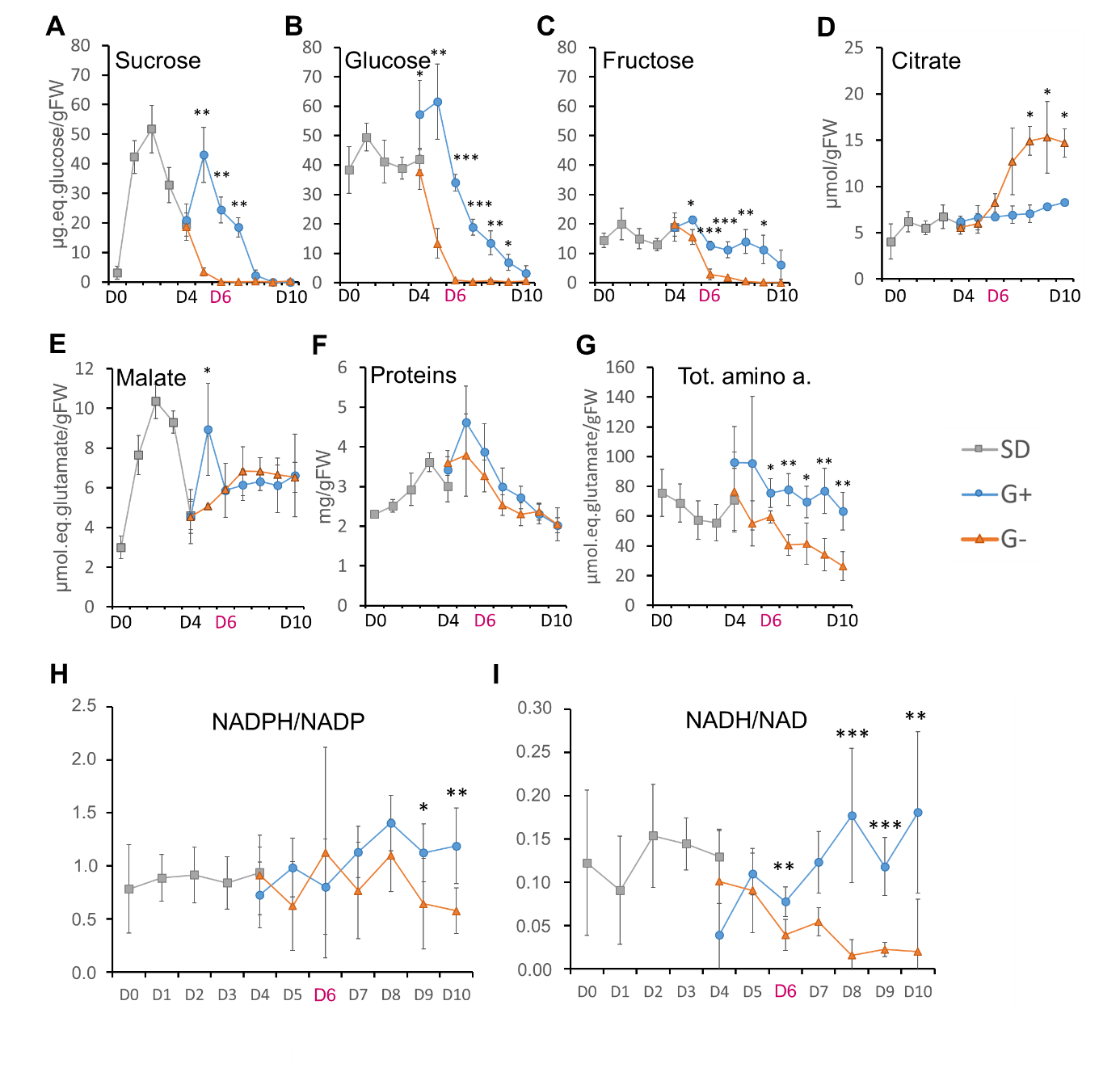


**Figure S5. Time-course of targeted metabolites accumulation and NAD(P)H/NAD(P) ratios in G- and G+ cells from D0 to D10.** **(A-C)** Accumulation of soluble sugars (measured in µq.eq.glucose/gFW), **(D, E)** organic acids, **(F)** total protein and **(G)** amino acids. **(H, I)** Time-course of NAD(P) oxidized and reduced form proportion through calculation of **(H)** NADPH/NADP and **(I)** NADH/NAD ratio in SD (grey), G+ (blue), G- (orange) conditions. Vertical bars represent CI (n=8). Stars indicate the level of significance of G+ and G- sample comparison (*p ≤ 0.05, **p ≤ 0.01, *** p ≤ 0.001, **** p ≤ 0.0001).


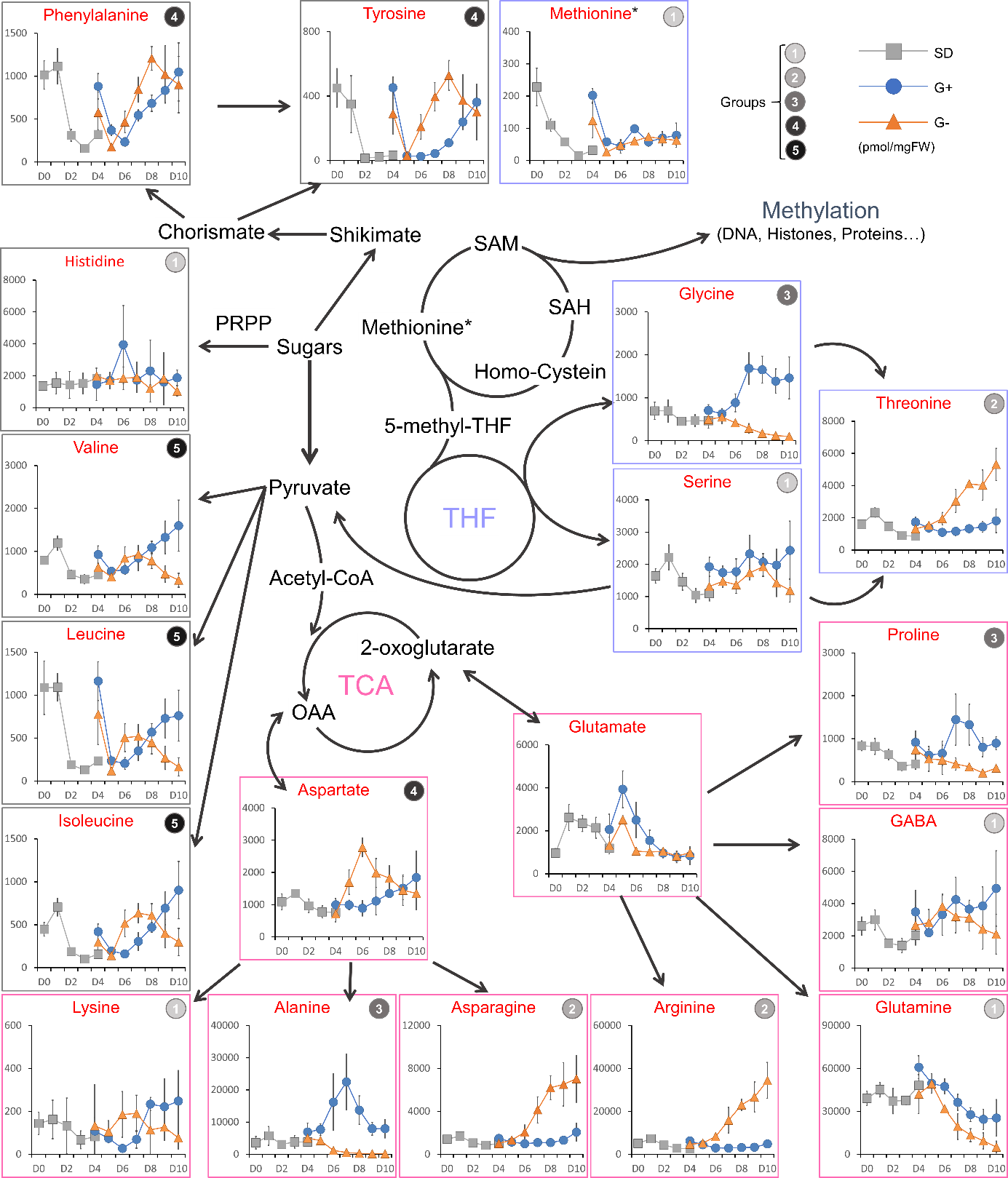


**Figure S6: Amino-acids accumulation (y-axis, pmol/mgFW) in grapevine cells harvested from D0 to D10 (x-axis) in standard (SD, grey), G+ (blue) and G- (orange) conditions.** Amino acids pathways are inferred according to the Kyoto Encyclopedia of Genes and Genomes (<https://www.genome.jp/kegg>) and published work about THF and Methionin cycle (Meng *et al.*, 2018). Vertical bars indicate CI (n=4). Amino acids were classified into 5 differents categories based on their accumulation profile (Supplementary information).

**Figure S7. Metabolic reorganization between G+ and G- cells. The flux map shows the differences in simulated fluxes between G- and G+ with increased fluxes in G- cells represented in orange, increased fluxes in G+ cells represented in blue, and unchanged fluxes in grey.** Fold-changes in flux intensities between G- and G+ were calculated as abs(log(abs(G-)/abs(G+), are represented by the width of the reaction arrows. The dotted lines represent fluxes that are directionally switched between G- and G+. Quantified metabolites are shown in big characters. Reversible reactions are represented by hexagons and irreversible reactions by diamonds. The direction of the arrows indicates the direction in which the reactions are written in the model. For the sake of clarity, side compounds were omitted. To facilitate interpretation, central carbon metabolism is highlighted in yellow, folate / SAM-SAH cycles in green and nucleotide metabolism in pink. Illustration designed with Cytoscape version 3.9.1
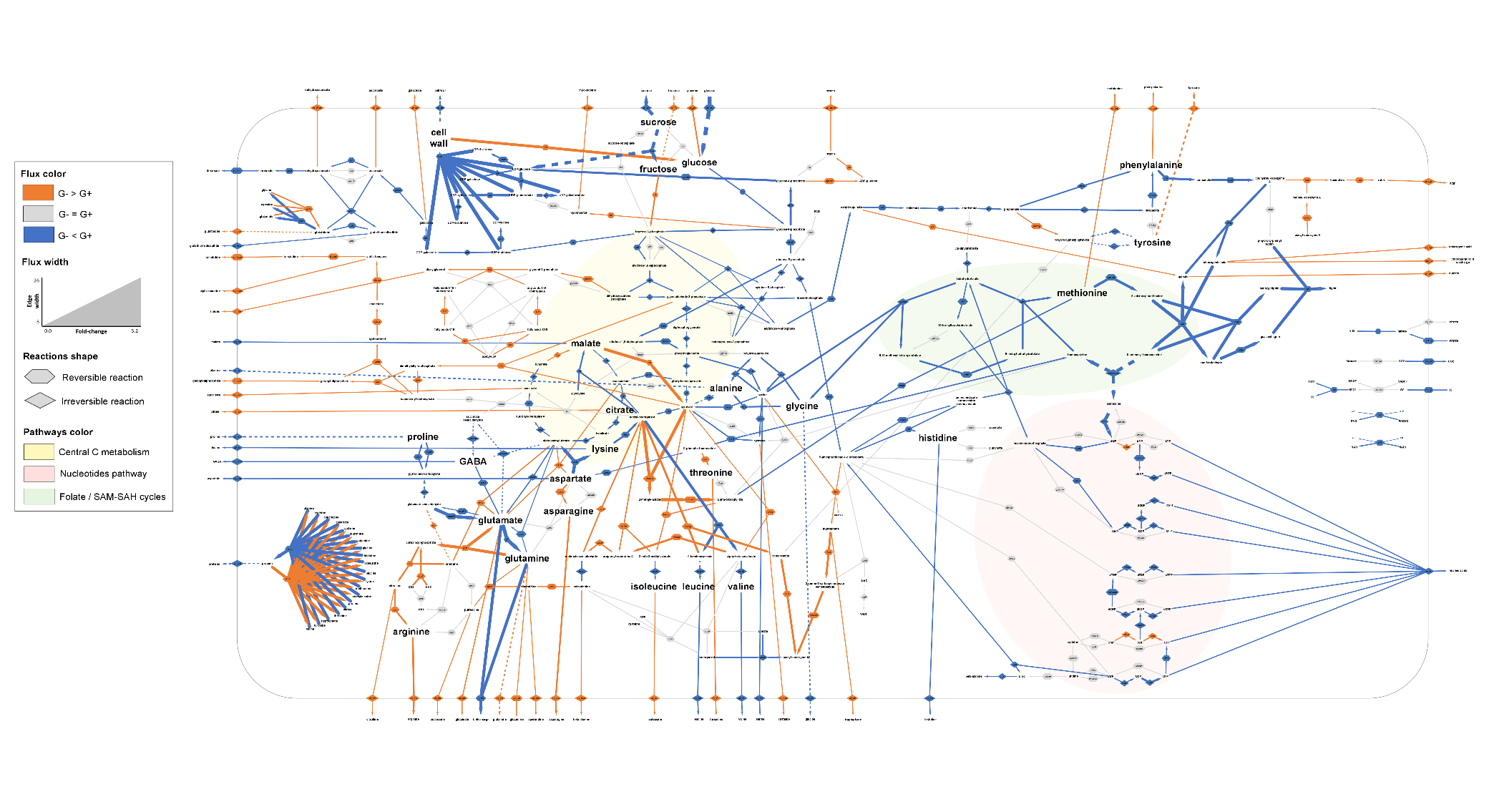


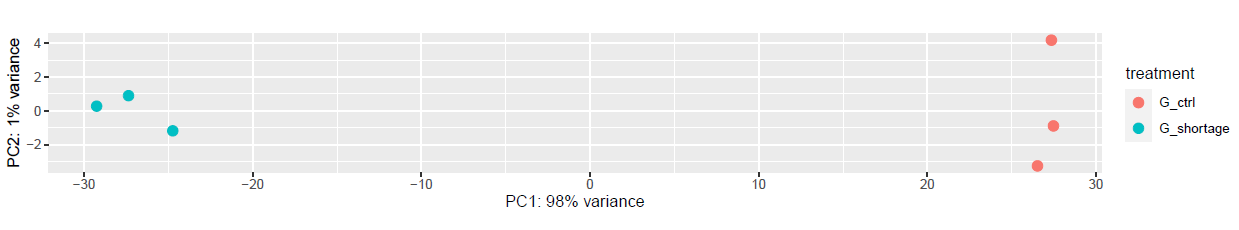


**Figure S8: Principal Component Analysis (PCA) of RNAseq profile of sequenced samples (n=3) at D6 in G+ and G- conditions. Variance explained by each PC is indicated in brackets.**


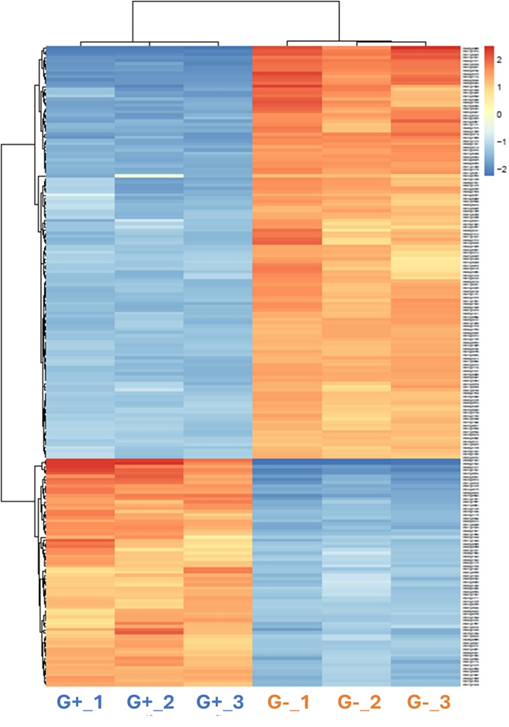


**Figure S9. Metabolic shift in G- cells is associated with a strong differential transcriptomic behavior.**

Heatmap representation of normalized expression of the 200 genes showing the highest variation within G+ and G- samples analyzed at D6 (pval <0.05). Colors represent the variability of gene expression among samples, with a normalization per row. Dendrogram were calculated using pearson’s correlation. G+: cells cultivated in glucose rich medium, G-: cells cultivated in glucose poor medium. Mapman visualization of identified DEGs in grapevine in G- cells at D6.


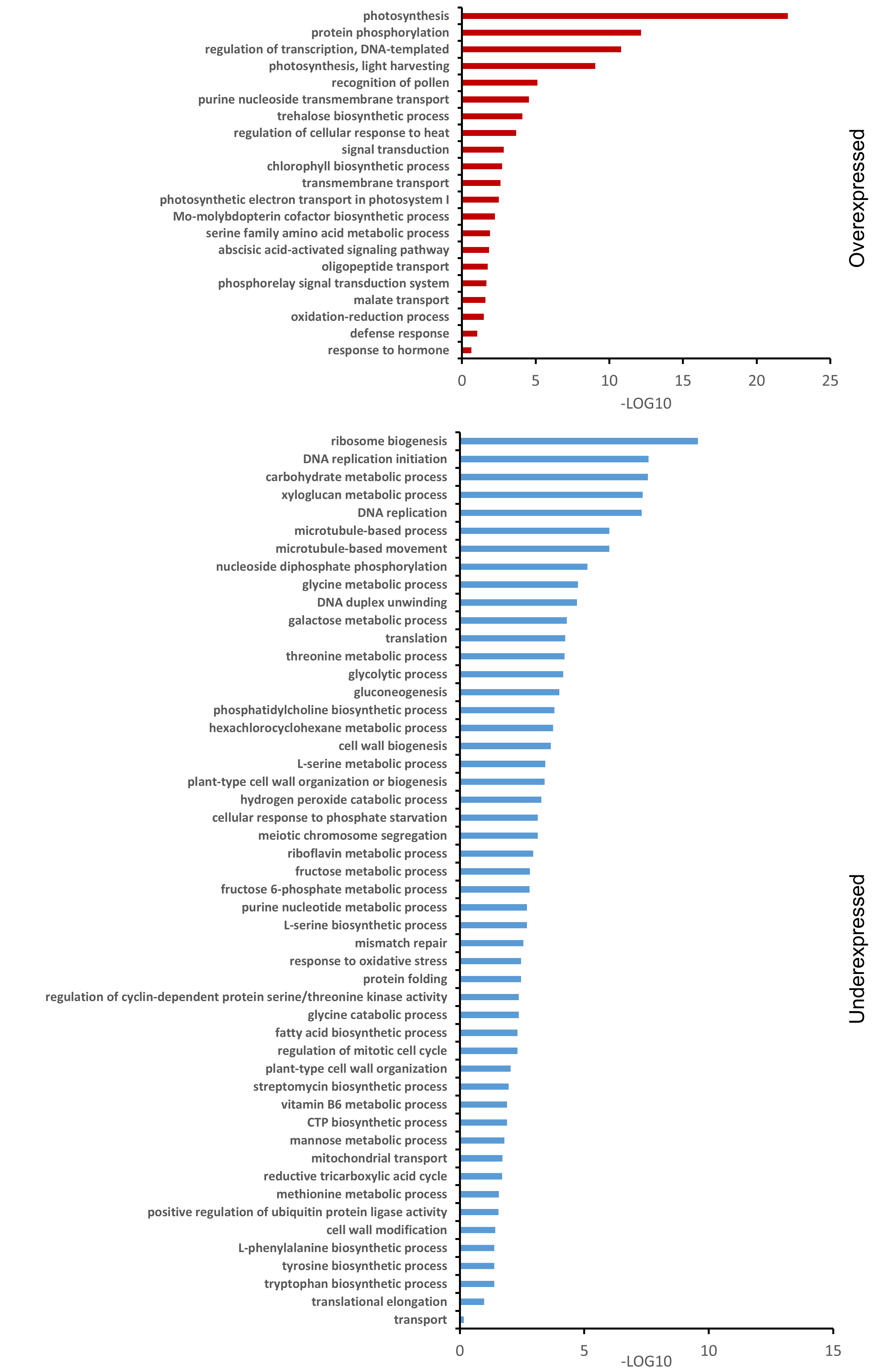


**Figure S10: Gene Ontology (GO) analysis performed on Differentially Expressed Genes (DEGs) identified by comparing G- and G+ conditions. Y-axis represent the biological process GO labels attributed to (A) overexpressed and (B) underexpressed genes. Enrichment values were normalized with the -LOG10 function.**


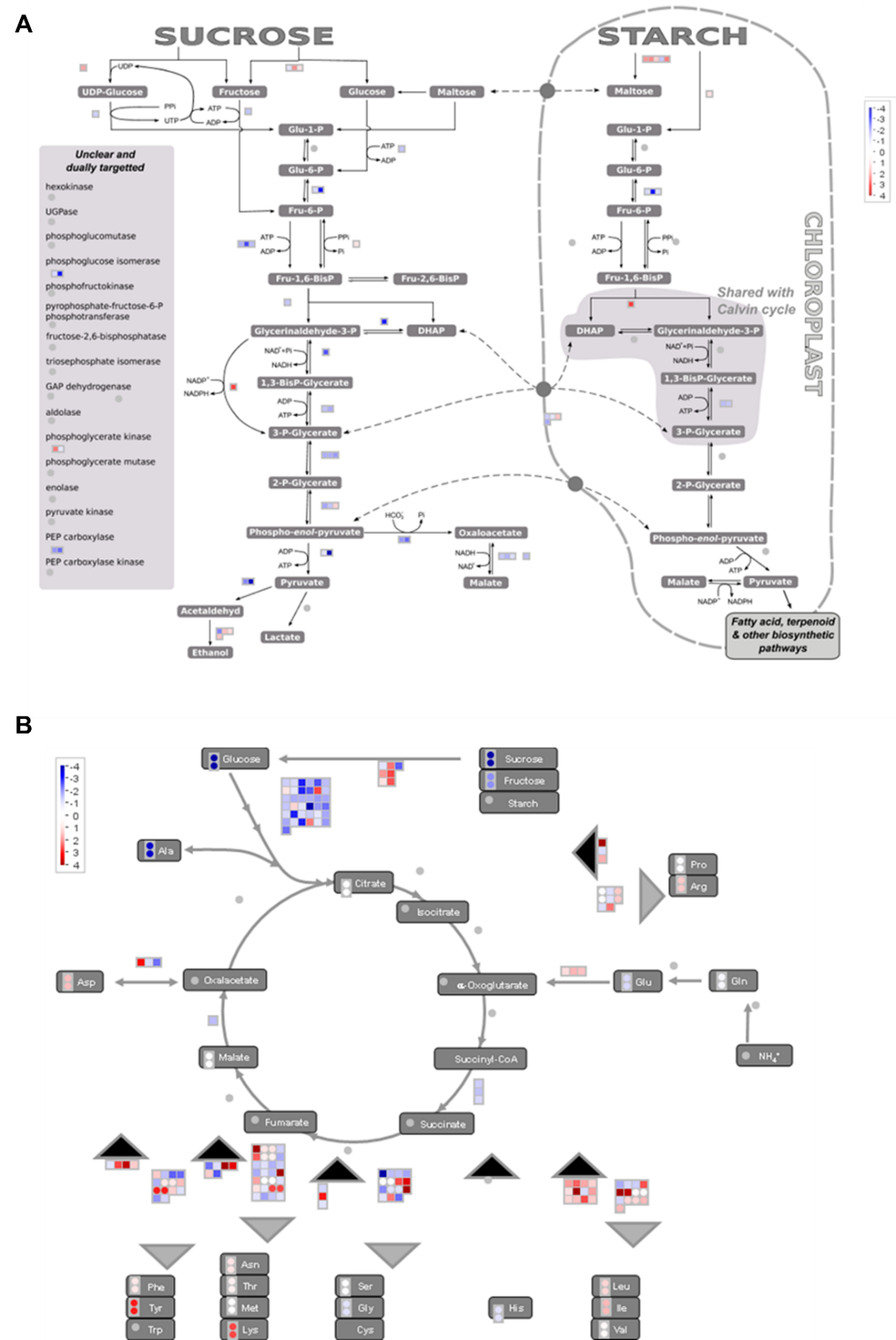


**Figure S11: Mapman view obtain from the DEGs between G- and G+ cells at D6. Visualization focused on glycolysis pathway (A) and TCA cycle (B). Each square represents a gene, each circle represents a metabolite. In red, genes/metabolites showing higher expression/accumulation in G- cells (devprived of sugar); in blue, genes/metabolites showing higher expression/accumulation in G+ cells (control conditions).**


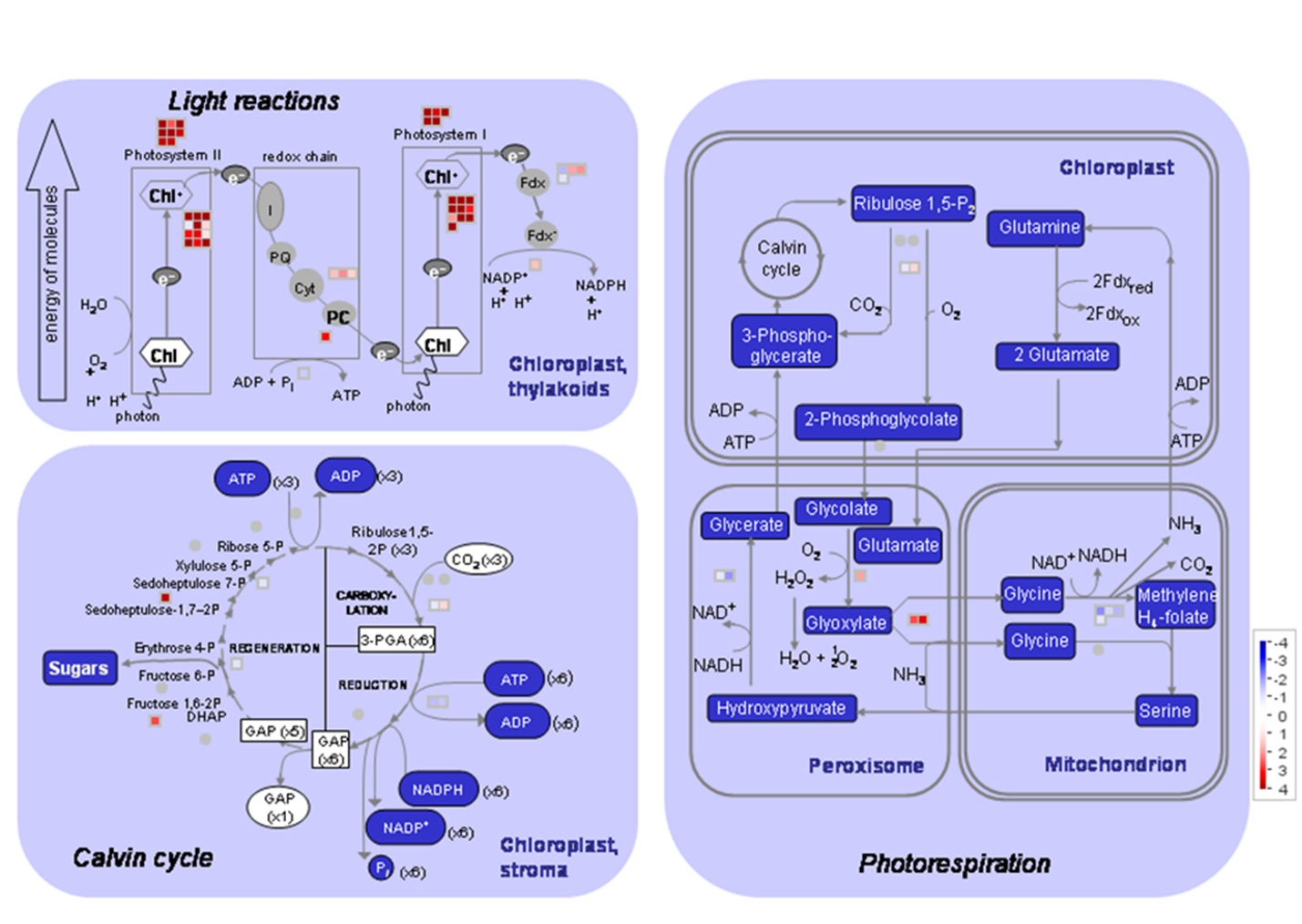


**Figure S12: Mapman representation of energy metabolism overview obtained from the DEGs identified in grapevine cells under carbon limitation conditions compared to control.**


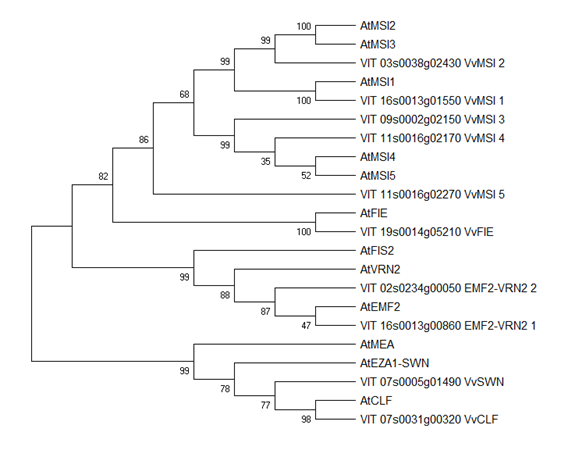


**Figure S13: Phylogenetic tree of PRC2 complex putative homologs identified in Vitis vinifera (Vv) and Arabidopsis thaliana (At). Tree generated with 1,000 bootstrap.**


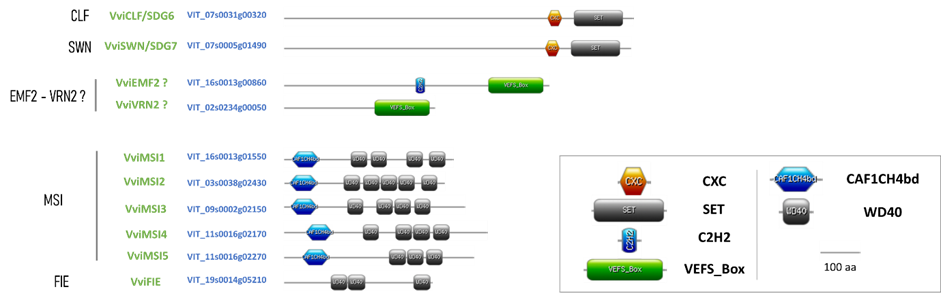


**Figure S14. PRC2 complex putative orthologs identified in grapevine their conserved domains.**


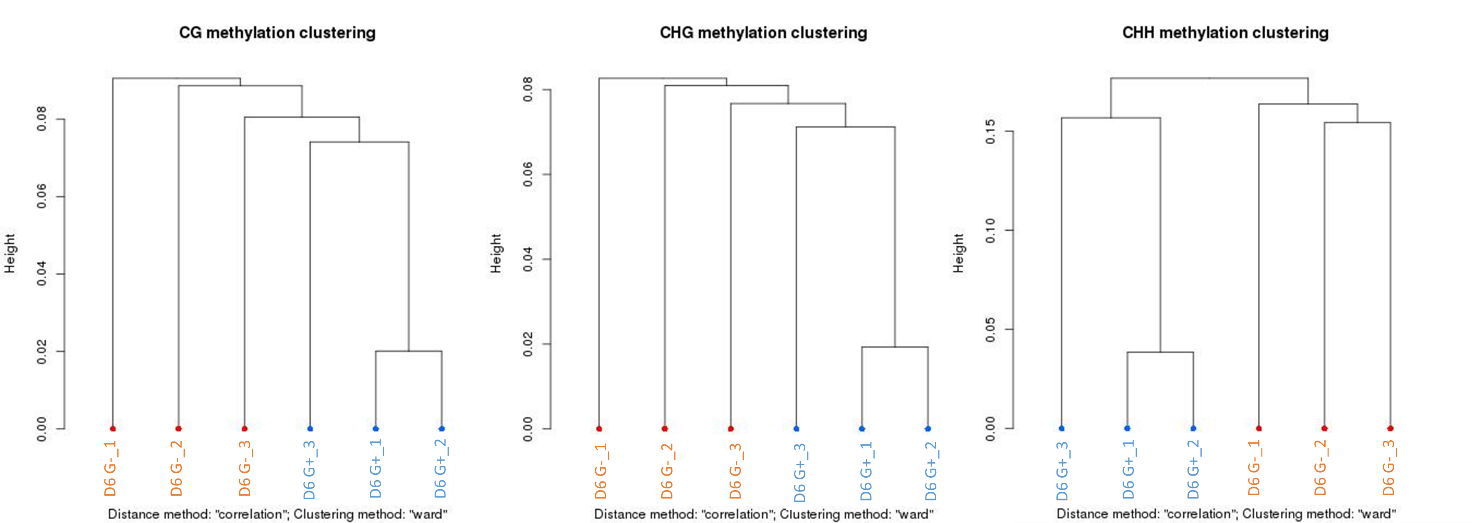


**Figure S15: Dendrogram representation of sample clustering based on their methylome profiles. Methylome analysis performed on G+ (blue) and G- (orange) at D6, clustering using ward method.**

**Supplementary Information References**

Clark, T.J., Guo, L., Morgan, J. and Schwender, J. (2020) ‘Modeling Plant Metabolism: From Network Reconstruction to Mechanistic Models’, *Annual Review of Plant Biology*, 71(1), pp. 303–326. Available at: https://doi.org/10.1146/annurev-arplant-050718-100221.

Colombié, S., Nazaret, C., Bénard, C., Biais, B., Mengin, V., Solé, M., Fouillen, L., Dieuaide‐Noubhani, M., Mazat, J., Beauvoit, B. and Gibon, Y. (2015) ‘Modelling central metabolic fluxes by constraint‐based optimization reveals metabolic reprogramming of developing *Solanum lycopersicum* (tomato) fruit’, *The Plant Journal*, 81(1), pp. 24–39. Available at: https://doi.org/10.1111/tpj.12685.

Holzhütter, H. (2004) ‘The principle of flux minimization and its application to estimate stationary fluxes in metabolic networks’, *European Journal of Biochemistry*, 271(14), pp. 2905–2922. Available at: https://doi.org/10.1111/j.1432-1033.2004.04213.x.

Lacrampe, N., Lugan, R., Dumont, D., Nicot, P.C., Lecompte, F. and Colombié, S. (2024) ‘Modelling metabolic fluxes of tomato stems reveals that nitrogen shapes central metabolism for defence against *Botrytis cinerea*’, *Journal of Experimental Botany*, p. erae140. Available at: https://doi.org/10.1093/jxb/erae140.

Meng, J., Wang, L., Wang, J., Zhao, X., Cheng, J., Yu, W., Jin, D., Li, Q. and Gong, Z. (2018) ‘METHIONINE ADENOSYLTRANSFERASE4 Mediates DNA and Histone Methylation’, *Plant Physiology*, 177(2), pp. 652–670. Available at: https://doi.org/10.1104/pp.18.00183.

Sweetlove, L.J., Obata, T. and Fernie, A.R. (2014) ‘Systems analysis of metabolic phenotypes: what have we learnt?’, *Trends in Plant Science*, 19(4), pp. 222–230. Available at: https://doi.org/10.1016/j.tplants.2013.09.005.

Xiao, W., Wang, R.-S., Handy, D.E. and Loscalzo, J. (2018) ‘NAD(H) and NADP(H) Redox Couples and Cellular Energy Metabolism’, *Antioxidants & Redox Signaling*, 28(3), pp. 251–272. Available at: https://doi.org/10.1089/ars.2017.7216.
